# Branched-chain amino acid assimilation enables mixotrophy of ammonia-oxidizing archaeal sponge symbionts

**DOI:** 10.1126/sciadv.aef9450

**Published:** 2026-09-04

**Authors:** Bettina Glasl, Katharina Kitzinger, Heidi M. Luter, Anton Legin, Stefan Schuster, Erika Salas, Nathalie Heldwein, Katarina Damjanovic, Marie Rutsch, Bram Vekeman, Nicole M. J. Geerlings, Petra Pjevac, Joana Séneca, Margarete Watzka, Wolfgang Wanek, Daan R. Speth, Michael Wagner

**Affiliations:** ^1^ Division of Microbial Ecology, Centre for Microbiology and Environmental Systems Science, University of Vienna, Vienna, Austria.; ^2^ Australian Institute of Marine Science, Townsville, Australia.; ^3^ Division of Terrestrial Ecosystem Research, Centre for Microbiology and Environmental Systems Science, University of Vienna, Vienna, Austria.; ^4^ Joint Microbiome Facility of the Medical University of Vienna and the University of Vienna, Vienna, Austria.; ^5^ Department of Chemistry and Bioscience, Aalborg University, Aalborg, Denmark.

## Abstract

Marine sponges and ammonia-oxidizing archaea (AOA) represent one of the earliest animal-microbe symbioses. AOA are considered metabolically constrained chemolithoautotrophs that remove nitrogenous waste within the sponge holobiont. Here, we expand this view by demonstrating that symbiotic AOA assimilate branched-chain amino acids (BCAA) as additional carbon and nitrogen sources. By combining stable isotope probing with fluorescence and chemical imaging, we trace the assimilation of ^13^C- and ^15^N-labeled BCAA (leucine, isoleucine, and valine) in the sponge holobiont *Ianthella basta* at single-cell resolution. We show that the ability to take up, degrade, and biosynthesize BCAA is a common adaptation among symbiotic AOA lineages. This ability may enable symbiotic AOA to modulate BCAA concentrations in their auxotrophic sponge hosts. Modulation of BCAA availability by symbionts may regulate the leucine-sensitive mTOR (mechanistic target of rapamycin) signaling pathway in sponges.

## INTRODUCTION

Sponges (Porifera) are one of the oldest extant animal phyla (*1*). These simple filter-feeding invertebrates have evolved in the Precambrian, long before the radiation of all other animal phyla (*2*). Sponges have successfully colonized virtually all aquatic habitats and contribute considerably to the biodiversity of our oceans (*3*). Their unparalleled filtration capacity makes them an important component of many marine ecosystems, where they play a crucial role in the recycling of dissolved organic matter (*4*). Many marine sponges are also known for their highly diverse and abundant microbiomes (*5*, *6*), whose metabolic functions underpin host health and resilience (*7*, *8*). The ancestral position of sponges in the evolution of animals and the symbiotic partnership with their metabolically complex microbiomes offer unprecedented insights into the early evolution and fundamental mechanisms of animal-microbe interactions.

Ammonia-oxidizing archaea (AOA) are one of the most widespread and abundant microbiome members of marine sponges (*9*, *10*). AOA symbionts, often vertically transmitted from the parents to the offspring, play a vital role in the recycling of nitrogenous waste products in the sponge holobiont and have been hypothesized to regulate signaling pathways of their sponge host via nitric oxide production (*11*–*17*). The symbiotic interaction of AOA with marine sponges is notable because AOA, one of the most successful microbial taxa in the global biosphere (*18*), rarely form symbiotic relationships with animals. However, sponge-associated AOA lineages have evolved multiple times within the order *Nitrososphaerales* (formerly known as *Thaumarchaeota*) and vary considerably in their host species specificity (*13*, *16*, *19*, *20*). Both free-living and sponge-associated AOA share the metabolic capacity to derive energy and reducing equivalents for the fixation of inorganic carbon from the oxidation of ammonia (NH_3_) to nitrite (NO_2_^−^). Genomes of sponge-associated AOA seem to support the utilization of alternative nitrogenous waste products for ammonia oxidation, such as urea, nitriles, cyanides, and creatinine (*13*, *15*, *16*), a genomic trait shared with free-living AOA (*9*). The utilization of alternative nitrogen sources (i.e., urea and cyanate) for ammonia oxidation has been proven for free-living AOAs (*21*, *22*); however, experimental evidence for such metabolic versatility in sponge-associated AOA is lacking. In contrast to most free-living AOA, sponge-associated AOA genomes also frequently encode for branched-chain amino acid (BCAA; i.e., valine, leucine, and isoleucine) transporters (*9*, *13*, *14*), which may represent a unique adaptation to their symbiotic lifestyle. Based on the presence of BCAA transporters in sponge-associated AOA genomes, it has been hypothesized that BCAA may serve as alternative carbon and nitrogen sources, thus classifying sponge-associated AOA as mixotrophs rather than strict chemolithoautotrophs (*13*).

Here, we test the hypothesis that sponge-associated AOA can assimilate BCAA as an alternative carbon and nitrogen source. Using the tropical marine sponge *Ianthella basta*, a well-established holobiont model with a highly stable and low-complexity microbiome (*23*), we investigated BCAA uptake by its dominant AOA symbiont, *Candidatus* Nitrosospongia ianthellae (*13*). We reconstructed the genomic potential for the transport, synthesis, and degradation of BCAA in both the sponge host and its AOA symbiont. To link genomic potential to activity, we quantified BCAA-derived ammonia oxidation, BCAA assimilation, and inorganic carbon fixation at bulk and single-symbiont cell levels. Our results demonstrate that BCAA are directly used as carbon and nitrogen source by the AOA symbiont *Ca.* N. ianthellae and likely other sponge-associated AOA lineages, confirming the unusual mixotrophic lifestyle of sponge-associated AOA. The ability to take up BCAA may play a critical role in host-symbiont communication by regulating the availability of BCAA, important signaling molecules and essential amino acids in animals.

## RESULTS

### Sponge-associated AOA genera are enriched in BCAA transporters

**Fig. 1.**
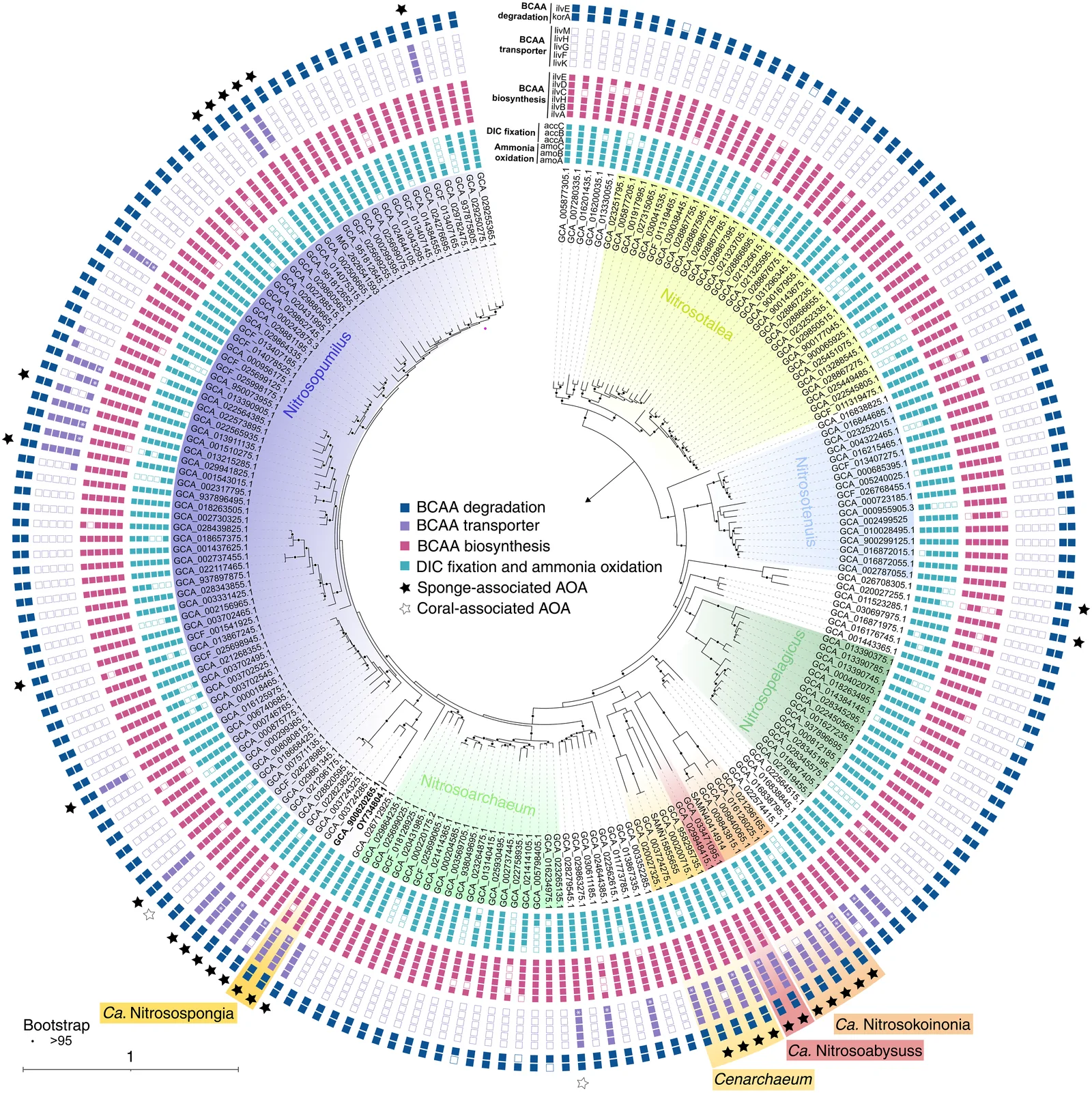
Phylogenomic tree of AOA. The maximum likelihood tree of AOA belonging to the family of *Nitrosopumilaceae* (included in GTDB version R220) is based on concatenated marker proteins. The tree highlights the presence of marker genes for ammonia oxidation (*amoABC*) and the autotrophic fixation of DIC [dissolved inorganic carbon as bicarbonate; (*96*)] via the 3-HP/4-HB cycle (*accABC*) in cyan. It further displays the phylogenetic distribution of the BCAA biosynthesis gene cluster (*ilvABHCDE*) in magenta, BCAA degradation genes (*ilvE* and *korA*) in blue, and the LIV-I BCAA transporter system (*livKFGHM*) in purple. Sponge- and coral-associated AOA lineages have evolved multiple times in the *Nitrosopumilaceae* family (indicated with black and white stars, respectively). Genomes of the sponge-specific AOA genera *Ca.* Nitrosokoinonia, *Ca.* Nitrosoabysuss, *Cenarchaeum*, and *Ca.* Nitrosospongia consistently encode for the LIV-I BCAA transporter. Ultrafast bootstrap (*n* = 1000) support is shown on the branches of the tree, and the scale bar corresponds to 1 estimated amino acid substitution per site. A white asterisk (*) inside a colored box indicates genes that were manually annotated.

We conducted a comparative analysis of all publicly available isolate genomes and metagenome-assembled genomes (MAGs; >80% completeness and <5% contamination) from the Genome Taxonomy Database (GTDB) version 220 (dereplicated at 95% average nucleotide identity) of AOA, including sponge-associated (*n* = 32), coral-associated (*n* = 2), and free-living (*n* = 166) species, to understand the phylogenetic distribution of BCAA biosynthesis and uptake capabilities across the archaeal family *Nitrosopumilaceae* (Fig. 1). Key genes for ammonia oxidation (*amoABC*), carbon fixation via the 3-hydroxypropionate/4-hydroxybutyrate (3-HP/4-HB) cycle (*accABC*), BCAA biosynthesis via the ilv operon (*ilvABHCDE*), and intracellular BCAA degradation including transamination (*ilvE*) and decarboxylation (*korA*) were encoded across all *Nitrosopumilaceae* lineages, irrespective of their native habitat. The four distinct sponge-specific AOA genera, *Cenarchaeum* (*20*), *Ca.* Nitrosokoinonia (*16*), *Ca.* Nitrosoabysuss (*19*), and *Ca.* Nitrosospongia (*13*), as well as several still unnamed sponge-associated AOA, additionally encode the high-affinity LIV-I system (*livKFGHM*), an adenosine 5′-triphosphate (ATP)–binding cassette (ABC) transporter specific for BCAA (Fig. 1) (*24*). Genes encoding for the LIV-I BCAA transport system were predominantly present in sponge-associated AOA (∼80% of all sponge-associated AOA genomes analyzed in this study). The majority (93%) of the here analyzed free-living AOA lack known BCAA transport systems; however, we did observe them in 12 genomes from sediment samples, especially from deep-sea and seep sites (representing ∼7% of all free-living AOA genomes analyzed in this study; see Fig. 1), which are often densely colonized by sponges (*25*).

To better understand the evolutionary origins of genes encoding the LIV-I BCAA transport system in *Nitrosopumilaceae*, we performed a phylogenetic assessment of the five subunits (fig. S1). Protein sequences of the LIV-I BCAA transport system appear to be monophyletic (exception: *livF* K01996), despite the fact that sponge-AOA symbioses have evolved multiple times and sponge symbionts are found in multiple lineages within the *Nitrosopumilacae* family. The phylogenetic neighborhood of the *livGFHM* genes (K01995-K01998) consists largely of other members of the *Thermoproteota*, including the members of the early-branching lineages of the putative chemoorganoheterotrophic *Nitrososphaerales* order that lack the genomic potential to oxidize ammonia and fix inorganic carbon (*26*). However, the phylogeny of these genes is inconsistent and additionally incongruent with (concatenated) marker gene phylogenies of the *Thermoproteota* phylum (figs. S1 and S2). In addition, the *livK* gene of the LIV-I BCAA transport system found in AOA genomes is closely related to *Bacteroidota* sequences and thus appears to have a different evolutionary history from the rest of the complex, despite occurring in an operon in the AOA. It thus seems likely that the *Nitrosopumilaceae* received four of their *liv* genes through horizontal gene transfer from another member of the *Thermoproteota* phylum and acquired *livK* separately. Subsequently, it is likely that several transfers within the *Nitrosopumilaceae* spread the *liv* genes to the distinct *Nitrosopumilaceae* lineages, including nearly all known sponge-associated AOA. Overall, the notable association of BCAA uptake systems with sponge-associated AOA indicates that this capability confers an important adaptation to their symbiotic lifestyle within sponges.

### Genomic potential of the *I. basta* sponge and its AOA symbiont to use BCAA

**Fig. 2.**
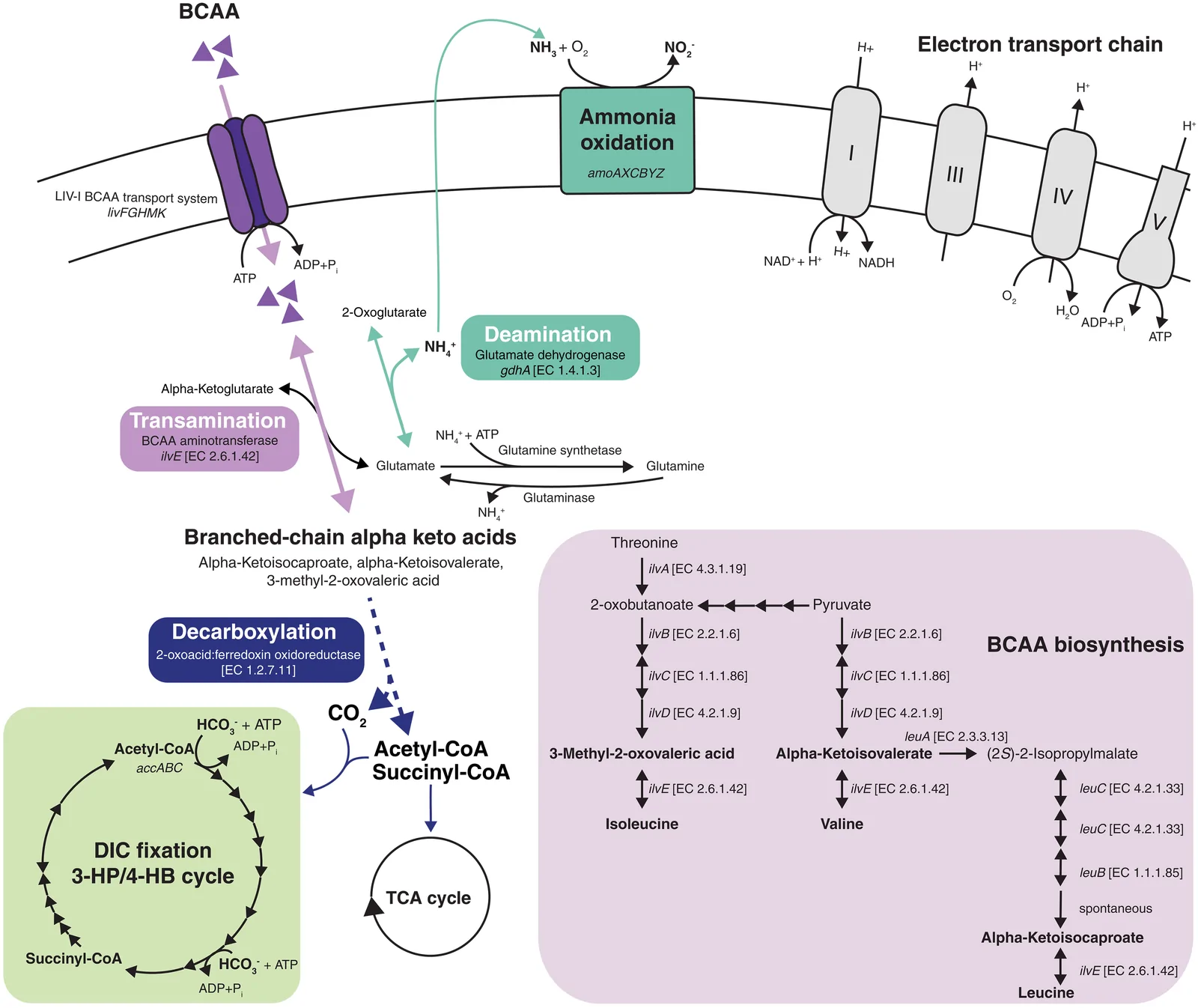
BCAA degradation and biosynthesis pathways of the AOA symbiont *Ca.* N. ianthellae. The *Ca.* N. ianthellae genome encodes for the high-affinity LIV-I BCAA (i.e., leucine, valine, and isoleucine) transport system. The first step in BCAA degradation is the transamination of BCAA with alpha-ketoglutarate. The enzyme BCAA aminotransferase (IlvE) catalyzes this reversible reaction, transferring an amino group from the BCAA to alpha-ketoglutarate and creating glutamate and the corresponding BCKA. Glutamate can then be used for protein synthesis, amino acid biosynthesis, and glutamine formation. Alternatively, glutamate can be deaminated by glutamate dehydrogenase (GdhA), releasing ammonium (NH_4_^+^), which can be used for ammonia oxidation. In the second step of the BCAA degradation, BCKA are decarboxylated either by an unknown branched-chain alpha keto acid dehydrogenase complex (BCKDC; not shown) or by 2-oxoacid:ferredoxin oxidoreductase, thereby releasing CO_2_, and producing acetyl-CoA and succinyl-CoA. These CoA conjugates can enter both the DIC fixation pathway and the tricarboxylic acid (TCA) cycle. In addition, *Ca.* N. ianthellae encodes all the hallmark genes characteristic of the chemolithoautotrophic lifestyle of AOA, including the ammonia-monooxygenase enzyme (AmoAXCBYZ), the electron transport chain (complex I-V), and the 3-HP/4-HB cycle for DIC fixation. The dashed line represents a hypothetical pathway.

To better understand the role of BCAA in the AOA-sponge symbiosis, we analyzed in detail the respective genomes of the AOA symbiont *Ca.* N. ianthellae (GCA_963457515.1) and its sponge host *I. basta* (GCA_964019415.1). The AOA symbiont *Ca.* N. ianthellae comprises on average 55.2% ± 10.6% (SD) of the *I. basta* microbiome (fig. S3) in our samples, based on 16*S* ribosomal RNA (rRNA) gene amplicon sequencing data. Its genome encodes for all hallmark genes for chemolithoautotrophic ammonia oxidation and inorganic carbon fixation (Fig. 2). In addition, the *Ca.* N. ianthellae genome encodes for the biosynthesis of BCAA (*ilv* and *leu* gene cluster) and the LIV-I BCAA transport system (Fig. 2). The latter has previously been shown to be expressed by *Ca.* N. ianthellae in the holobiont using metaproteomics (*13*). Furthermore, *Ca.* N. ianthellae has the genomic potential to perform the first step of the BCAA degradation pathway, the transamination of BCAA using a BCAA aminotransferase (*ilvE*), resulting in the production of glutamate and branched-chain keto acids (BCKAs). Deamination of glutamate using the encoded glutamate dehydrogenase (*gdhA*) leads to the production of ammonium and thus is an alternative source of the substrate for the energy metabolism of this AOA. The second step of the BCAA degradation pathway is the decarboxylation of the BCKA, catalyzed in other organisms by the BCKA dehydrogenase complex (EC 1.2.4.4) or by the BCKA ferredoxin reductase (EC 1.2.7.7). Homologous genes encoding for both enzymes are missing from the *Ca.* N. ianthellae genome. However, the *Ca.* N. ianthellae genome encodes for a member of the multienzyme family 2-oxoacid:ferredoxin oxidoreductase (Fig. 2). Enzymes of this family mediate the decarboxylation of pyruvate, 2-oxoglutarate, and BCKA to their corresponding acyl–coenzyme A (CoA) derivatives (*27*–*29*), indicating that 2-oxoacid:ferredoxin oxidoreductase may be involved in BCAA degradation in *Ca.* N. ianthellae and other AOA (Fig. 1).

In contrast to its AOA symbiont and the other two dominant heterotrophic symbionts, *Ca.* Luteria ianthellae and *Ca.* Taurinisymbion ianthellae (*23*), the sponge *I. basta* lacks the genomic repertoire for BCAA biosynthesis but has the complete set of genes encoding for the BCAA degradation pathway (fig. S4A). Furthermore, the *I. basta* genome also encodes for and transcribes the conserved BCAA-stimulated mechanistic target of rapamycin (mTOR) pathway of eukaryotes (fig. S4B), including the ancestral leucine-sensing mechanism LARS1 (leucyl-tRNA synthetase 1) known to regulate mTORC1 (mTOR complex 1) activity (*30*, *31*). The mTORC1 pathway in sponges remains understudied, but it has been shown that mTORC1 plays an important role in integrating symbiont-derived nutrients into host metabolism and homeostasis in cnidarians (*32*), deep-sea mussels (*33*), and aphids (*34*). The effect of BCAA on the microtubule organization via mTORC1 (fig. S4B) may also have a vital role in the functioning and formation of ciliated cell types in sponges such as the choanocytes, a highly proliferative cell type that contributes to tissue regeneration and sexual reproduction and is specialized to capture food particles (*35*, *36*). Overall, our analysis suggests that *I. basta* is auxotrophic for BCAA and requires the active uptake of BCAA, yet further investigations are needed to better understand the regulatory role of BCAA on the mTORC1 pathway. As such, the ability of its AOA and the two other dominant symbionts to modulate the BCAA availability in the sponge holobiont may play a fundamental role in the host-symbiont communication.

### BCAA use by the *I. basta* sponge holobiont

The *I. basta* pore water contained substantial concentrations of free BCAA compared to total free amino acid concentrations typically found in seawater (0.001 to 1.4 μmol N liter^−1^) (*37*)—2.19 ± 0.38 (SD) μM, 1.97 ± 0.75 (SD) μM, and 0.81 ± 0.13 (SD) μM of valine (Val), leucine (Leu), and isoleucine (Ile), respectively, measured in field control samples (fig. S5A). This indicates that both the sponge and its symbionts are consistently exposed to BCAA, which may serve as potential substrates. To test whether BCAA are actively metabolized by the holobiont, we conducted 24-hour incubation experiments using an equimolar mix of fully ^13^C- and ^15^N-labeled Val, Leu, and Ile (1 μM total BCAA concentration) in parallel incubations containing either sponge explants or filtered seawater (fig. S6).

Over the course of the incubation, the labeled BCAA were progressively depleted from the incubation water in both setups, but with faster consumption observed in incubations containing sponge explants [nonsignificant decrease of labeled BCAA in filtered seawater over the first 12 hours of incubation of 0.01 ± 0.01 (SE) μM hour^−1^ (*P* = 0.059), compared with a significant decrease of 0.04 ± 0.004 (SE) μM hour^−1^ (*P* = 0.001), in incubations containing sponge explants; see also fig. S5B]. Correspondingly, the sponge holobiont biomass showed slight enrichment in both ^13^C and ^15^N after 24 hours compared to nonincubated sponge field controls (fig. S5C), indicating active assimilation of BCAA-derived carbon and nitrogen by the holobiont. Alongside BCAA depletion, we detected increasing levels of dissolved ^13^C-labeled CO_2_ in the incubation seawater, reaching up to 4 μM (fig. S5D). Given that complete respiration of the added BCAA would produce ∼5.6 μM ^13^C-CO_2_, this suggests that a substantial fraction (∼70%) of the supplied BCAA was respired.

**Fig. 3.**
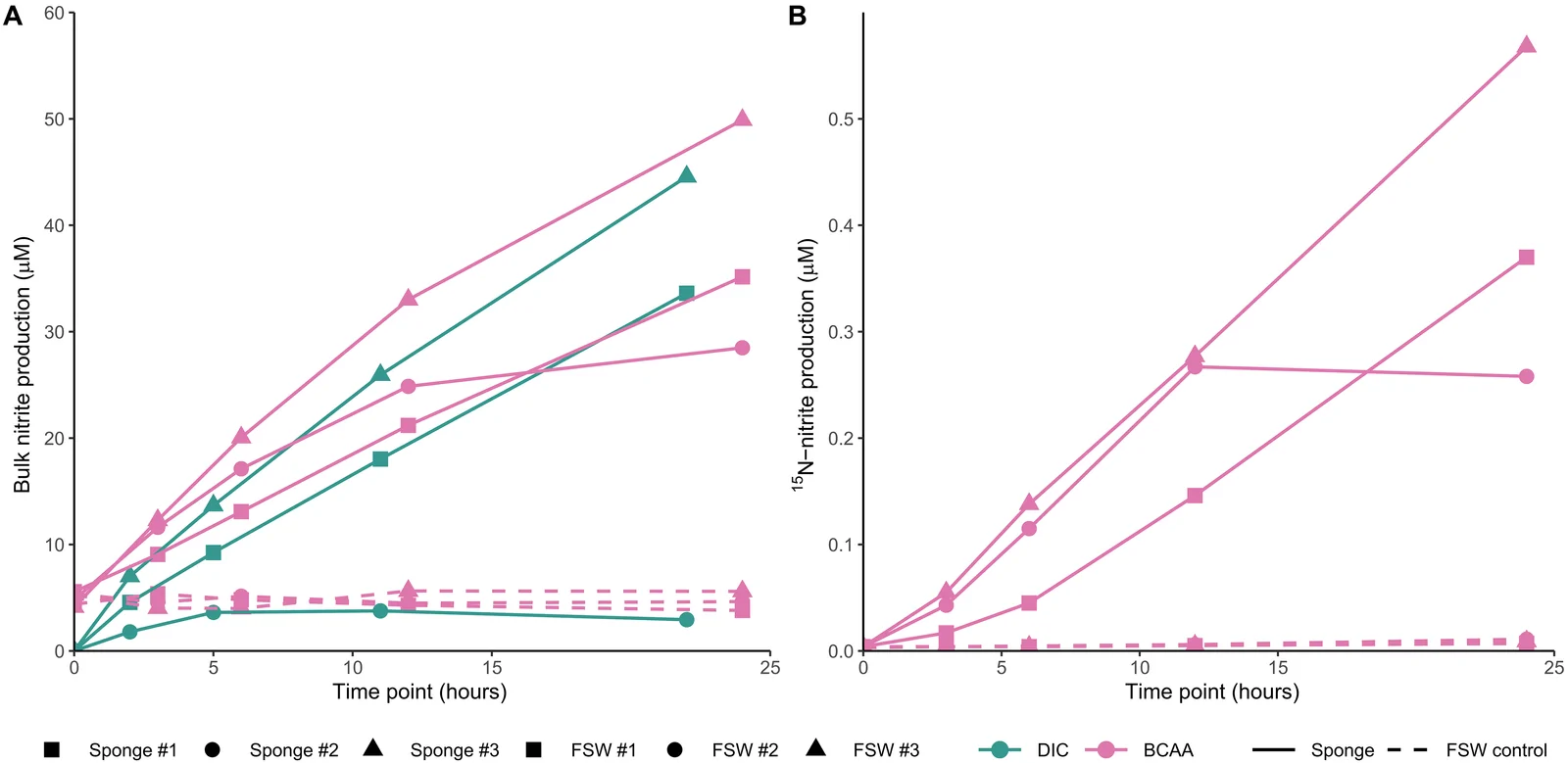
Nitrification activity of the *I. basta* sponge holobiont. (**A**) Bulk nitrite (NO_2_^−^) production (in micromolar) in sponge containing and filtered seawater control (FSW) jars in the ^13^C-labeled DIC and ^13^C^15^N-labeled BCAA incubation experiments. No significant bulk nitrite formation was detected in the seawater control (nitrite formation normalized to the sponge wet weight is provided in fig. S7A). (**B**) ^15^N-nitrite (^15^N-NO_2_^−^) production (in micromolar) in sponge and FSW jars in the ^13^C^15^N-labeled BCAA incubation experiment. Sponge explants were incubated in filtered seawater amended with an equimolar mix of ^13^C^15^N-labeled valine, leucine, and isoleucine. Bulk and ^15^N-nitrite production rates normalized to the sponge wet weight are shown in fig. S7B.

A first indication that sponge-associated AOA contribute to the observed BCAA uptake was the detection of ^15^N-nitrite. *I. basta* contains no nitrite-oxidizing symbiont (*13*); therefore, the nitrite produced by AOA from the oxidation of nitrogenous substrate does not get further oxidized to nitrate. We observed significant and linear production of both isotopically unlabeled and ^15^N-labeled nitrite in the incubations containing sponge explants [bulk nitrite formation of 1.353 ± 0.169 (SE) μM hour^−1^ (*P* = 1.41 × 10^−6^) corresponding to 0.170 ± 0.026 μmol gWW^−1^ hour^−1^ (*P* = 0.0036); ^15^N-nitrite formation of 16.9 ± 2.2 (SE) nM hour^−1^ (*P* = 1.72 × 10^−6^) corresponding to 2.13 ± 0.32 (SE) nmol gWW^−1^ hour^−1^ (*P* = 7.34 × 10^−6^); rates calculated across biological triplicates] (Fig. 3 and fig. S7). In contrast, we observed no significant isotopically unlabeled nitrite production and only minute ^15^N-labeled nitrite in incubations containing seawater only [0.22 ± 0.03 (SE) nM hour^−1^, rates calculated across biological triplicates; Fig. 3]. This proves that the symbiotic AOA of *I. basta* both were metabolically active and oxidize BCAA-derived ^15^N-labeled ammonia, with up to 56% of the provided ^15^N from BCAA being recovered as ^15^N-nitrite.

However, these bulk analyses cannot differentiate between direct and indirect use of BCAA for nitrification by the symbiotic AOA. The heterotrophic bacterial symbionts, *Ca.* L. ianthellae and *Ca.* T. ianthellae, of the *I. basta* holobiont have the genetic capacity for BCAA degradation (*23*) and thus can release ^15^N-labeled ammonium, which can subsequently be oxidized to ^15^N-nitrite by the symbiotic AOA.

### Symbiotic AOA cells assimilate both BCAA and ^13^C-CO_2_

To determine whether *Ca.* N. ianthellae is capable of directly taking up BCAA and using these amino acids for growth, we measured ^13^C- and ^15^N-BCAA assimilation into single symbiont cells. To achieve this, we specifically visualized the three dominant symbionts of *I. basta*, the AOA symbiont (*Ca.* N. ianthellae), the alphaproteobacterial symbiont *Ca.* L. ianthellae, and the gammaproteobacterial symbiont *Ca.* T. ianthellae, using catalyzed-reporter deposition fluorescence in situ hybridization (CARD-FISH) in combination with 4′,6-diamidino-2-phenylindol (DAPI) staining, and measured cellular isotopic enrichment by nanoscale secondary ion mass spectrometry (NanoSIMS). While isotopic enrichment was negligible in the measured sponge biomass, all symbiont groups were significantly enriched in both ^13^C and ^15^N after 24-hour incubation with ^13^C^15^N-labeled BCAA. This experimental evidence is consistent with the genomic capacity of all three main symbionts to import and use BCAA.

**Fig. 4.**
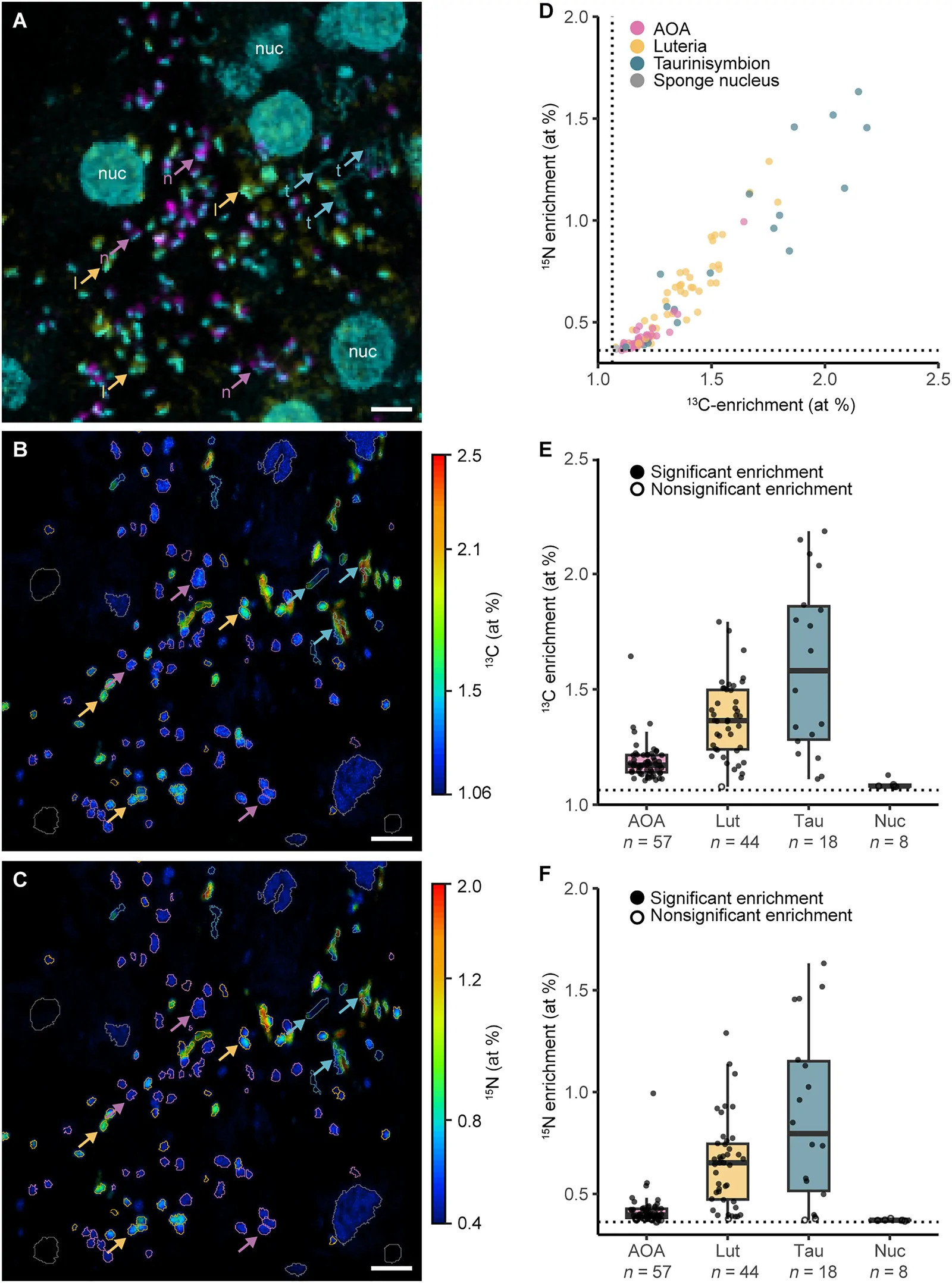
Single-cell ^13^C and ^15^N-assimilation from BCAA measured by NanoSIMS. Correlative imaging of the same cryosection position was done using CARD-FISH and NanoSIMS. (**A**) CARD-FISH image counterstained with DAPI, visualizing the AOA symbiont *Ca.* N. ianthellae (n, magenta, stained by probe Arch915), the alphaproteobacterial symbiont *Ca.* L. ianthellae (l, yellow, stained by probe AlfD729), the gammaproteobacterial symbiont *Ca.* Taurinisymbion ianthellae (t, cyan, counterstained with DAPI), and sponge nuclei (nuc, cyan, counterstained with DAPI) in a 5-μm cryosection of the *I. basta* holobiont. (**B** and **C**) Corresponding NanoSIMS images of ^13^C enrichment and ^15^N enrichment after the incubation with isotopically labeled BCAA. Quantified regions of interest of the different cell types are marked by colored outlines (*Ca.* N. ianthellae in magenta, *Ca.* L. ianthellae in yellow, *Ca.* T. ianthellae in cyan, and sponge nucleus and tissue marked in gray). Scale bars, 2 μm. The saturation intensity (hue) of isotope enrichment was modulated by the measured phosphorus signal for better visibility. (**D**) Correlation between ^13^C and ^15^N single cell isotopic enrichment from ^13^C^15^N-labeled BCAA incorporation. (**E** and **F**) Single cell ^13^C and ^15^N enrichment of the main *I. basta* microbial symbiont groups (AOA = *Ca.* N. ianthellae, Lut = alphaproteobacterial *Ca.* L. ianthellae, and Tau = gammaproteobacterial *Ca.* T. ianthellae) and sponge nuclei (Nuc). Boxplots depict the 25 to 75% quantile range, with the center line depicting the median (50% quantile); whiskers encompass data points within 1.5× the interquartile range. Dotted lines in (D) to (F) indicate average natural ^13^C and ^15^N abundance values as measured for symbiont cells in nonincubated sponge biomass (1.06 at % ^13^C and 0.36 at % ^15^N, respectively). The total number of measured cells (*n*) within each group is indicated below the *x*-axis labels. Individual fluorescence channels used for CARD-FISH detection and the raw NanoSIMS images are provided in fig. S8.

The highest isotopic enrichment for both ^13^C and ^15^N was observed in the gammaproteobacterial symbiont cells [1.60 ± 0.37 atomic % (at %) ^13^C and 0.88 ± 0.43 at % ^15^N, mean ± SD], followed by alphaproteobacterial symbiont cells (1.37 ± 0.16 at % ^13^C and 0.65 ± 0.22 at % ^15^N, mean ± SD) and the AOA symbiont cells (1.19 ± 0.08 at % ^13^C and 0.42 ± 0.09 at % ^15^N, mean ± SD; natural abundance values were 1.06 ± 0.01 at % ^13^C and 0.36 ± 0.007 at % ^15^N, mean ± SD across all cells) (Fig. 4 and fig. S8). This pattern may reflect generally higher cellular activity in the heterotrophic gamma- and alphaproteobacterial symbionts compared to symbiotic AOA *Ca.* N. ianthellae. The mean ratio of new carbon to new nitrogen assimilation, considering significantly enriched cells, was similar for the gammaproteobacterial and alphaproteobacterial symbionts [10.5 ± 8.8 (SD) and 10.1 ± 6.8 (SD) fmol C cell^−1^ day^−1^ to fmol N cell^−1^ day^−1^, respectively], whereas the symbiotic AOA *Ca.* N. ianthellae assimilated significantly more carbon relative to nitrogen [16.9 ± 5.5 (SD) fmol C cell^−1^ day^−1^ to fmol N cell^−1^ day^−1^; nonparametric Kruskal Wallis test; Kruskal-Wallis chi-square = 29.5, df = 2, *P* = 3.9 × 10^−7^; Dunn’s post hoc test with Holm adjustment: AOA–alphaproteobacterial symbiont: *P*_adj_ = 1.5 × 10^−6^, AOA–gammaproteobacterial symbiont: *P*_adj_ = 2.7 × 10^−4^].

The consistent enrichment in both ^13^C and ^15^N of the symbiotic AOA *Ca.* N. ianthellae provides strong evidence for their direct BCAA utilization. ^13^C-DIC (dissolved inorganic carbon) derived from the heterotrophic breakdown of BCAA would be diluted in the large ambient DIC pool with natural ^13^C isotope abundance (seawater contains ∼2.3 mM DIC; we anticipate similar concentrations in the sponge mesohyl). Thus, only minimal ^13^C labeling of *Ca.* N. ianthellae from inorganic carbon fixation due to cross-feeding would be expected. Likewise, ^15^N-ammonium released from heterotrophic breakdown of ^13^C^15^N-labeled BCAA would also be diluted into the ambient unlabeled ammonium pool (from, e.g., organic matter breakdown by the sponge and/or its symbionts; fig. S9), lowering the likelihood of ^15^N cross-feeding from BCAA.

To date, autotrophic inorganic carbon fixation by *Ca.* N. ianthellae has been inferred from the correlation between nitrification activity and bulk ^13^C-DIC uptake by the sponge holobiont (*13*), as well as from the presence of the 3-HP/4-HB carbon fixation pathway in their genomes and in the metaproteome (*13*, *23*). However, single-cell evidence for autotrophic carbon fixation by *Ca.* N. ianthellae within the sponge holobiont has been lacking. To test whether *Ca.* N. ianthellae cells also actively fix inorganic carbon, in addition to utilization of BCAA, we conducted parallel incubations with ^13^C-DIC and measured isotope incorporation at the single-cell level using NanoSIMS. In these incubations, as in the BCAA incubations, sponge-associated AOA were metabolically active, as evidenced from increasing nitrite concentrations (Fig. 3 and fig. S7), and *Ca.* N. ianthellae fixed significantly more ^13^C-DIC than other microbial cells in the holobiont (one-sided Mann-Whitney *U* test, *W* = 14,007, *P* < 2.2 × 10^−16^; fig. S10A).

Using the measured carbon enrichment from both BCAA and DIC for *Ca.* N. ianthellae, we estimated single-cell C-based growth rates in our incubations to be 0.011 ± 0.007 (SD) day^−1^ and 0.018 ± 0.010 (SD) day^−1^, respectively. These growth rates correspond to doubling times of ∼95 and ∼55 days, respectively, from BCAA and DIC. Such long doubling times are unusual compared to other marine AOA, e.g., 0.7 days in pure culture (*38*) to several days for free-living marine AOA in the eutrophic Gulf of Mexico (*39*). The slow growth rates observed here may indicate that sponge-associated AOA require additional C sources to CO_2_ and BCAA. However, the slow growth rates observed here may also reflect the specific incubation conditions, the dilution of cellular carbon by the CARD-FISH procedure (see Materials and Methods), or alternatively, an inherently slow turnover of the AOA population within the sponge holobiont. Nevertheless, our measurements demonstrate that BCAA assimilation can contribute substantially (∼58% of autotrophic carbon fixation) to *Ca.* N. ianthellae growth in the sponge holobiont under our incubation conditions (fig. S10B). Notably, this estimate assumes that symbiont cells are exposed both to the isotopic background of the incubation seawater at the start of the incubation and of the native BCAA in sponge pore water. As the experimentally added isotopically labeled BCAA were depleted over the course of the incubation, this represents a conservative estimate. An even more conservative assumption, assuming that symbionts do not experience unlabeled pore-water BCAA but only the labeled BCAA provided, still yields BCAA-derived growth rates equivalent to ∼10% of autotrophic carbon fixation–derived growth (fig. S10B).

Together, our results show that the AOA symbiont *Ca.* N. ianthellae assimilates both organic carbon and nitrogen deriving from BCAA in the sponge holobiont, in addition to autotrophic carbon fixation, thereby supporting a mixotrophic lifestyle. Beyond benefiting from the assimilation of organic compounds to enhance growth rates, the AOA *Ca.* N. ianthellae symbiont cells potentially regulate host physiology through BCAA level modulation.

## DISCUSSION

AOA are generally considered to be chemolithoautotrophs, yet there has been a long-standing debate over whether they are obligate autotrophs or can grow mixotrophically (*40*–*44*). The observation that alpha-keto organic acids, such as pyruvate, stimulated the growth of some AOA pure cultures (*45*, *46*) initially suggested a mixotrophic lifestyle of some of these ammonia-oxidizer strains. However, later, it was found that alpha-keto organic acids are not assimilated as a carbon source during ammonia oxidation but instead stimulate AOA growth via the nonenzymatic detoxification of H_2_O_2_ (*47*), which can inhibit catalase-negative AOA. Amino acid use by AOA has also been tested extensively. While pure cultures of the marine AOA *Nitrosopumilus adriaticus* and *Nitrosopumilus piranensis*, which do not encode a BCAA uptake system, did not use leucine as a source of energy and/or carbon (*48*), earlier studies showed incorporation of label from a mixture of 15 tritiated amino acids (*49*) and from tritiated leucine (*50*), respectively, into marine free-living AOA cells (not yet recognized as such in those studies) using microautoradiography-FISH. Despite the fact that some marine free-living AOA are encoding BCAA uptake systems (Fig. 1), the isotope incorporation observed in these former studies could also stem from cross-feeding from other community members that were the primary users of the labeled amino acids. Two more recent single-cell analysis studies of environmental free-living marine AOA communities using a ^13^C^15^N-labeled algal amino acid mixture showed primarily use of amino acid–derived nitrogen (likely largely via cross-feeding) (*51*, *52*). In these studies, only a small subset of the analyzed AOA cells were enriched in both ^15^N and ^13^C, and always with much less per cell C assimilation compared to N (*51*, *52*). Taken together, amino acid labeling experiments with marine free-living AOA demonstrated some activity, but direct evidence for mixotrophic growth of these nitrifiers is still limited.

Here, we show the simultaneous and consistent assimilation of ^13^C and ^15^N from BCAA by an AOA, by analyzing the sponge-associated AOA *Ca.* N. ianthellae in situ using correlative CARD-FISH–NanoSIMS imaging (Fig. 4). We also experimentally demonstrate the capacity of *Ca.* N. ianthellae for fixation of DIC (fig. S10A) and thus provide compelling evidence for a mixotrophic lifestyle of these ammonia oxidizers in the sponge holobiont. As de novo biosynthesis of BCAA is energetically costly for cells, ranging from 10 ATP per molecule of valine to four ATP per molecule of leucine and three ATP per molecule of isoleucine (*53*), the ability to take up BCAA from the environment, in addition to their de novo biosynthesis, might confer a selective advantage for sponge-associated AOA lineages. This is particularly relevant given that BCAA concentrations in the sponge pore water samples (fig. S5) exceeded those previously reported for seawater by two to three orders of magnitude (*54*). Simultaneous capability for BCAA uptake via the LIV-I transport system and autotrophic carbon fixation via the 3-HP/4-HB cycle appears to be a widespread adaptation among sponge-associated AOA (Fig. 1) and is likely a fundamental adaptation to their symbiotic lifestyle. Despite the independent evolution of distinct sponge-associated AOA lineages, subunits of the BCAA LIV-I transporter system are of monophyletic origin (figs. S1 and S2). Whether the uptake of BCAA LIV-I transporter subunits into sponge-associated AOA genomes stems from multiple instances of horizontal gene transfer or is a result of joint loss by most free-living AOA cannot be irrefutably determined from our analyses. However, the monophyly of the AOA BCAA transporter genes points to a single acquisition within the AOA. As all BCAA LIV-I transporter–containing AOA share a common ancestor within the *Nitrosopumilaceae*, the distribution of the transporter can be explained by one acquisition and multiple losses. However, the BCAA gene phylogenies are incongruent with the genome phylogeny. Notably, the deepest branching BCAA transporter–containing clade in the genome phylogeny (DRGT01) is among the most derived groups in the gene phylogenies. Consequently, this pattern is more consistent with a single ancestral acquisition followed by frequent horizontal gene transfer among AOA lineages. While the few free-living AOA genomes that encode a LIV-I BCAA transport system (e.g., deep-sea sediment clade DRGT01) share an evolutionary origin with the sponge-associated AOA, gene phylogenies indicate that the uptake of LIV-I BCAA genes seems to have originally occurred in a sponge-associated AOA.

**Fig. 5.**
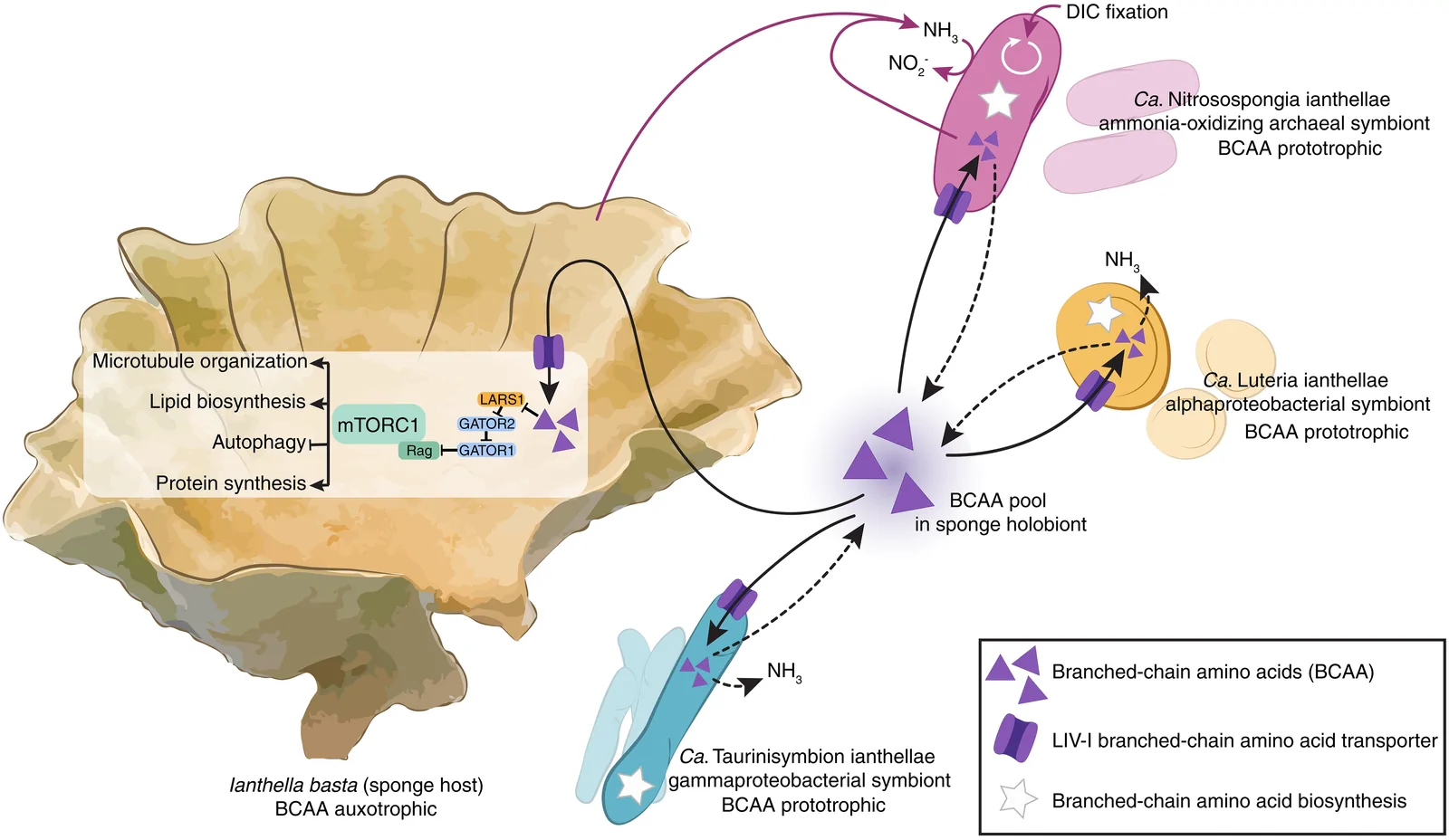
Schematic overview of BCAA biosynthesis and assimilation capabilities in the *I. basta* holobiont. The three dominant symbionts of the sponge *I. basta*, the archaeal *Ca.* N. ianthellae, the alphaproteobacterial *Ca.* L. ianthellae, and the gammaproteobacterial *Ca.* T. ianthellae, take up BCAA (valine, leucine, and isoleucine) as carbon and nitrogen sources from the environment (exogenous supply of BCAA). Additionally, all three symbiont genomes have a full set of genes that encode BCAA biosynthesis. Unlike the two heterotrophic bacterial symbionts, the AOA symbiont can also fix DIC autotrophically with energy derived from the oxidation of ammonia (NH_3_), a metabolic waste product of the sponge host and a product of BCAA catabolism. Thus, the AOA symbiont derives its carbon for growth from autotrophic and heterotrophic modes of nutrition (mixotrophy). The BCAA pool in the sponge holobiont is potentially fueled by exogenous BCAA uptake, the phagocytosis of microbial cells, and the BCAA biosynthesis of symbiont cells. The sponge host itself lacks the genomic repertoire for BCAA biosynthesis and therefore relies on the uptake of these essential amino acids from the BCAA pool. BCAA are known to stimulate the highly conserved mTORC1 signaling pathway in other animals, which is fully encoded and transcribed in the *I. basta* genome; thus, BCAA regulation by sponge symbionts may play a crucial role in host-microbe interactions in this basal metazoan phylum.

The sponge *I. basta*, like all animals, is auxotrophic for BCAA and relies on the uptake of exogenous BCAA (fig. S4). The readily available BCAA pool in *I. basta* can derive from various sources, such as phagocytosis of symbionts, the secretion of BCAA by symbionts into the mesohyl, and from the surrounding environment via intercellular gaps between sponge cells (*55*) or the engulfment of dissolved organic matter via pinocytosis (*56*, *57*). Similar to its AOA symbiont, we were able to show that also the heterotrophic symbionts of *I. basta* take up exogenously supplied BCAA as a carbon and nitrogen source (Fig. 4), despite being genomically equipped to biosynthesize their own BCAA (*23*). Thus, all three symbionts of *I. basta* have the ability to modulate BCAA concentrations in the sponge mesohyl, potentially regulating the availability of essential amino acids in their auxotrophic host (Fig. 5). Furthermore, BCAA, such as leucine, are also known regulators of the mTORC1 pathway, a conserved signaling pathway in eukaryotes (*30*). Besides its central role in cell proliferation, autophagy, and protein synthesis, mTORC1 also has a functional role in various nutritional symbioses, including the coral-algal (*32*), the aphid-*Buchnera* (*34*), and the mussel-bacteria symbioses (*33*). In the latter, mTORC1 regulates phagosome digestion of symbiosomes in *Bathymodiolus* mussels in response to the nutrient supply from the symbionts (*33*). However, whether mTORC1 plays a similar role in sponges and whether microbial symbionts actively influence this central eukaryotic signaling pathway by altering BCAA levels and thus modulating the ancestral LARS1 leucine-sensing mechanisms remains unknown. Future studies targeting the interaction between symbiont metabolism, BCAA availability, and host mTOR pathways will be key to gain insights into the regulatory mechanisms in sponge holobionts.

## MATERIALS AND METHODS

### Phylogenetic distribution of BCAA uptake and biosynthesis genes in the AOA family *Nitrosopumilaceae*

MAGs (dereplicated at 95% average nucleotide identity, >80% completeness, and <5% contamination) of the family *Nitrosopumilaceae* included in the GTDB version R220 (*58*) and publicly available MAGs of sponge-associated AOA (dereplicated at 99% average nucleotide identity, with >80% completeness and <5% contamination) were used for the comparative genome analysis. Completeness and contamination were assessed using checkM v1.2.3 (*59*). In total, 200 MAGs were included in the analysis, of which 32 MAGs originated from marine sponges and two MAGs from corals. Concatenated amino acid sequences of marker proteins were obtained using checkM v1.2.3 (*59*) and used for phylogenomic reconstruction with IQ-TREE v2.4.0 (*60*) using the best-fit model (Q.insect+I+R8) according to the Bayesian Information Criterion (BIC) and 1000 ultrafast bootstraps. Genomes of isolates from the terrestrial AOA sister family *Nitrososphaeraceae* (*Nitrososphaera viennensis* and *Nitrososphaera gargensis*) were used as the outgroup. The phylogenetic tree was subsequently visualized in iTOL v7 (*61*), highlighting sponge- and coral-associated AOA symbiont lineages, the presence of genes encoding for BCAA degradation (transamination and decarboxylation), LIV-I BCAA transporter, BCAA biosynthesis, and key genes for ammonia oxidation (*amoABC*) and carbon fixation (*accABC*) annotated using eggnog mapper v2.1.10 (*62*).

Proteins annotated as K01995 (*livG*), K01996 (*livF*), K01997 (*livH*), K01998 (*livM*), and K01999 (*livK*), corresponding to the five subunits of the LIV-I BCAA transport system (*63*), were retrieved from the GlobDB release 220 (*64*). Length distribution of the annotated proteins was assessed using “seqkit watch” (*65*), and likely false positives were removed by restricting the hits to proteins of length ranges 230 to 280 (K01995), 220 to 280 (K01996), 275 to 320 (K01997), 260 to 460 (K01998), and 350 to 470 (K01999) amino acids. False negatives in the KEGG annotation, and proteins in genomes from GlobDB r226 that had not been included yet in GlobDB r220, were subsequently added by using each curated protein set as seed in a protein alignment score ratio analysis against the GlobDB release 226 (*64*), resulting in final datasets of 371,412 (K01995), 439,115 (K01996), 413,331 (K01997), 393,125 (K01998), and 585,274 (K01999) amino acid sequences. Sequence datasets were clustered at 50% sequence identity (with the exception of K01999, which was clustered at 40% identity) using usearch v12.0 (*66*) and aligned using muscle5 v5.3 (*67*), and trees were calculated using FastTree2 v2.1.11 (*68*).

The presence of AOA BCAA genes in the gene phylogenies described above was assessed in iToL v7 (*61*). For all subunits, the AOA-derived genes clustered in a single clade of the full phylogeny. There were several singleton genes that clustered outside this clade of interest. We interpret these singleton genes as arising from binning errors in the genomes included in the GlobDB. For the clade of interest of each gene, trees containing all sequences, without prior identity clustering, were calculated as described above. For three subunits (K01995, K01997, and K01998), the AOA-derived genes formed a monophyletic clade, whereas for the remaining two subunits (K01996 and K01999), two distinct clades were observed.

We used the 3584 *Thermoproteota* genomes and 52 *Korachaeota* genomes in the GlobDB (*64*) to generate a concatenated marker gene phylogeny. Therefore, HMM hits for 76 archaeal marker genes were exported from the anvi’o contigs databases available for the GlobDB using anvi-get-sequences-for-hmm-hits (as integrated in anvi’o v9), using the --concatenate-genes flag to align each of the marker genes using muscle v3.8.1551 (*67*) and concatenate the resulting alignments. A phylogeny based on the resulting alignment was calculated using FastTree2 v2.1.11 (*68*).

### Metabolic reconstruction of *Ca.* N. ianthellae and *I. basta*

The closed genome of the AOA symbiont *Ca.* N. ianthellae (GCA_963457515.1) was annotated using eggnog mapper v2.1.10 (*62*). Annotations were used to reconstruct the following key metabolic pathways: ammonia oxidation, respiratory electron transport, inorganic carbon fixation via the 3-HP/4-HB cycle, biosynthesis of BCAA, BCAA transport, and BCAA degradation. The *livK* gene (K01999), encoding for the substrate binding subunit of the LIV-I BCAA transporter, was not annotated when using eggnog-mapper. We therefore screened the protein sequences of the *Ca.* N. ianthellae genome for LIV-K family (PF13458) proteins using the hmmscan function of HMMER v3.4 (hmmer.org). The corresponding *livK* gene was identified to be located in an operon with the other LIV-I transporter encoding genes in the *Ca.* N. ianthellae genome.

The *I. basta* genome [GCA_964019415.1; 215M base pairs (bp), haploid number = 5, chromosome number = 16, genome completeness = 71.6%] has been previously sequenced and annotated using RNA sequencing data with BRAKER2 (*69*) by the Aquatic Symbiosis Genomics Project (*70*). The data are available at the Aquatic Symbiosis Genomics Portal (https://portal.aquaticsymbiosisgenomics.org/data/root/details/Ianthella%20basta). The protein fasta file was submitted to the GhostKOALA platform for automatic KO assignment and KEGG pathway reconstruction (*71*). The completeness of the BCAA biosynthesis pathway, the BCAA degradation pathway, and the mTOR pathway was assessed, and pathway maps were reconstructed using KEGG Mapper.

### Sponge collection

Three individuals of the sponge species *I. basta* (yellow, thin morphotype) were collected from Davies Reef (18°49′19.1″S 147°38′11.3″E, Great Barrier Reef, Australia) at 11m depth in December 2022. Sponges were transferred to the flow-through aquaria system at the National SeaSimulator at the Australian Institute of Marine Science (Townsville, Australia). After a 2-week acclimation period, each sponge individual (*n* = 3) was fragmented into three equal-sized explants of the fan-shaped sponge body (∼5 cm × 7 cm) using sterile scissors. One of the three explants per sponge individual (*n* = 3) was subsampled for 16*S* rRNA gene amplicon sequencing and BCAA pore water concentration measurements. The other two explants of each sponge individual (total *n* of explants = 6) were kept in an outdoor flow-through aquaria system (2500 liters) under natural lighting and ambient seawater temperatures (∼28.5°C) for 1 week prior to further experiments. An additional *I. basta* individual was collected at the same sampling site in March 2024 as a natural abundance control and for 16*S* rRNA gene amplicon sequencing. All samples were collected under the permit G21/38062.1 issued by the Great Barrier Reef Marine Park Authority.

### Stable isotope incubation experiments of *I. basta* explants

Custom-made incubation jars (*n* = 9; fig. S6) were acid-washed using 1 M HCl, rinsed, filled with 800 ml of 0.2 μm filter–sterilized seawater (Sterivex, Merck Pty Ltd., Darmstadt, Germany), and sealed to avoid exchange with the atmosphere. Six of the jars were amended with an isotopically labeled BCAA mix (1 μM final total BCAA concentration, with l-valine–^13^C_5_^15^N, l-leucine–^13^C_6_^15^N, and l-isoleucine–^13^C_6_^15^N each at 0.33 μM concentration; all Sigma-Aldrich, Saint Louis, USA) and 5 μM of ^14^NO_2_^−^ (Carl Roth, Karlsruhe, Germany) as a trap for any produced ^15^N-NO_2_^−^ from BCAA-derived ammonia oxidation. The remaining three jars were amended with 500 μM NaH^13^CO_3_ (DIC; Sigma-Aldrich, Saint Louis, USA). One explant from each sponge individual (*n* = 3) was placed in one of the two different stable isotope experiments: (i) BCAA incubation and (ii) DIC incubation. The three remaining jars amended with ^13^C^15^N-BCAA were used as seawater control incubations. All incubation jars (*n* = 9) were randomly distributed in a chest-type orbital shaker incubator (Thermoline Scientific), which was set to 80 rpm and 28.5°C, matching the in situ seawater temperature. All jars were kept in the dark throughout the experiment. Oxygen concentrations were monitored throughout the experiments via oxygen sensor spots and the FireStingGo2 pocket oxygen meter (PyroScience GmbH, Aachen, Germany). Pure oxygen gas was added with a syringe and needle to the headspace of the incubation jars containing sponge explants to maintain 100% oxygen saturation throughout the 24-hour incubation period.

Water samples (10 ml each) from all incubation jars (*n* = 9) were collected at five time points (see fig. S6) throughout the incubation experiments via the sampling port septum using a needle and a syringe (jars remained closed throughout the experiment). The collected water samples were sterile filtered (Minisart PES filter, 0.2 μm, VWR, Vienna, Austria) and used to determine nitrification activity (stored at −20°C), BCAA concentrations (BCAA experiment only, stored at −75°C), ^13^C enrichment of DIC (stored at 4°C in headspace-free glass vials closed with rubber septa, Labco, Lampeter, UK), and NH_4_^+^ concentrations (stored at −20°C; see fig. S6). After the last sampling time point, the wet weight of each sponge explant was measured. Sponge explants of the BCAA experiment were washed three times for 20 min in 0.2 μm filter-sterilized seawater (Sterivex, Merck Pty Ltd., Darmstadt, Germany) at 80 rpm at 28.5°C to remove excess isotopically labeled BCAA from the tissue. The tissue was then briefly rinsed with 0.2 μm filter-sterilized seawater and subsampled for DNA extractions, CARD-FISH–NanoSIMS imaging, bulk stable isotope enrichment analysis, and measurement of sponge pore water concentrations of BCAA. The remaining incubation water in the jars (∼760 ml) was filtered onto 0.2 μm Sterivex filters (Merck Pty Ltd., Darmstadt, Germany) for subsequent DNA extractions (*n* = 3 for BCAA seawater control samples, *n* = 1 for sponge incubations).

### ^13^C-DIC measurements in incubation seawater samples

^13^C-DIC production from ^13^C^15^N-labeled BCAA was assessed by conversion of all DIC to CO_2_ by acidification and subsequent measurement of ^13^C/^12^C ratios and at % ^13^C in CO_2_ by gas chromatography–isotope ratio mass spectrometry (GC-IRMS). Briefly, 12-ml glass vials (Labco, Lampeter, UK) were amended with 25 μl of 20% phosphoric acid (Sigma-Aldrich, Darmstadt, Germany), closed airtight with a screw cap containing a septum, and flushed with He for 3 min. Then, 0.5 ml aliquots of incubation water samples (prefiltered through a 0.2 μm PES filter) were injected into the closed, flushed glass vials, carefully swirled to mix with phosphoric acid, and incubated overnight before measurement of the CO_2_ released into the headspace using a gas preparation system connected to the GC-IRMS (GasBench II connected to Delta V Advantage IRMS, Thermo Fisher Scientific, Bremen, Germany). ^13^C/^12^C ratios in CO_2_ were calibrated against reference CO_2_ standards and freshly prepared standards of NaHCO_3_ (natural abundance).

### BCAA concentrations in sponge pore-water and incubation seawater samples

Valine, leucine, and isoleucine concentrations were measured in sponge pore water samples (*n* = 9) and in incubation water samples (*n* = 30; prefiltered through a 0.2 μm PES filter) of the BCAA incubation experiment. Sponge pore water was recovered by placing fresh sponge fragments into 0.5 ml plastic centrifuge tubes with perforated bottoms (five holes punched with a 23-gauge needle, Braun, Melsungen, Germany). The perforated tubes were placed into 2 ml plastic collection tubes, and *I. basta* pore water was collected via centrifugation at 1000*g* for 1 min. The freshly collected incubation water was filtered through a 0.2 μm syringe filter (CHROMAFIL, Macherey Nagel, Düren, Germany). All samples were immediately frozen and stored at −75°C until further analysis.

Prior to analysis, pore water samples (*n* = 9) were diluted 1:10 in artificial seawater (per liter: 26.4 g NaCl, 6.8 g MgSO_4_·7H_2_O, 5.7 g MgCl_2_·6H_2_O, 1.47 g CaCl_2_·2H_2_O, 0.66 g KCl, 0.2 g KH_2_PO_4_, and 0.19 g NaHCO_3;_ all Sigma-Aldrich, Darmstadt, Germany), and any remaining debris was pelleted by centrifugation at 10,000*g* for 5 min. BCAA concentrations were quantified against the respective standards (0.0625 to 2 μM) prepared in artificial seawater. Samples and standards were derivatized with the AccQ-Tag Ultra Derivatization Kit (Waters, Milford, MA, USA) and subsequently measured on a UPLC-Orbitrap mass spectroscopy system (*72*) with modifications to the UPLC settings. In brief, samples were measured using a UPLC Ultimate 3000 system (Thermo Fisher Scientific, Bremen, Germany) linked to an Orbitrap Q Exactive HRMS system (Thermo Fisher Scientific, Bremen, Germany) with an electrospray ionization (H-ESI) source. Chromatographic separation was conducted on a Water AccQ-Tag Ultra C18 column (2.1 mm × 100 mm, 1.7 μm particles; Milford, MA, USA) with an ACQUITY column in-line guard filter (2.1 mm, 0.2 μm; Milford, MA, USA) at 55°C column temperature. The mobile phase consisted of 0.1% formic acid in MilliQ (eluent A) and 0.1% formic acid in acetonitrile (eluent B). The following solvent gradient was applied: 0 to 0.5 min kept constant at 0.1% B, 0.5 to 2.5 min increased to 5% B, 2.5 to 8 min increased to 20% B, 8 to 8.25 min increased to 90% B, 8.25 to 11 min maintained at 90% B, 11 to 11.2 min decreased to 0.1% B, and 11.2 to 14.5 min maintained at 0.1% B for column re-equilibration. The flow rate was set to 0.4 ml min^−1^, and the injection volume was 1 μl. The Orbitrap Q Exactive HRMS system was operated using full-mass scan mode (mass/charge ratio of 150 to 1000) in the ESI positive mode. The automatic gain control (AGC) target values were set to 3 × 10^6^, and the resolution was set to 70,000. The lock mass correction was set to 214.0896 (contaminant from plasticizer). The spray voltage was 3.5 kV, while the capillary temperature was set to 300°C. The sheath gas consisted of 35 arbitrary units, and the auxiliary gas of 15 arbitrary units.

### Bulk nitrification and ^15^N-nitrite formation from BCAA

Bulk nitrite and nitrate production in the incubations was analyzed photometrically using the Griess protocol (*73*, *74*). Measured concentrations were converted to nitrite production rates per sponge wet weight by accounting for the removal of incubation water aliquots during sampling.

^15^N-nitrite production from ^13^C^15^N-BCAA was analyzed by conversion of nitrite to nitrous oxide using azide (*75*) and subsequent measurement of ^15^N-N_2_O to ^14^N-N_2_O ratios using GC-IRMS. Briefly, sample aliquots (500 μl) were incubated at 37°C and 100 rpm shaking with 100 μl of 1 M NaN_3_ in 5% acetic acid and 500 μl of 1 M HCl for 24 hours. The reaction was stopped by injecting 100 μl of 6 M NaOH. For ^15^N-N_2_O measurement, the samples were purged with helium gas at 10 ml min^−1^, passed through a magnesium perchlorate and ascarite trap to remove water vapor and CO_2_, and cryofocused in two consecutive liquid nitrogen traps (first trap of stainless steel and second trap of fused silica, Finnigan Precon, Thermo Fisher Scientific, Bremen, Germany). The cryofocused sample gas was released at room temperature and transferred through a Nafion trap into a GC column (GS-G, Gasbench II head space analyzer, Thermo Fisher Scientific, Bremen, Germany) to separate N_2_O from other gases and finally injected into the ion source of the IRMS instrument (Finnigan Delta Advantage V, Thermo Fisher Scientific, Bremen, Germany).

### 16*S* rRNA gene amplicon sequencing and data analysis

DNA from (i) field-control sponge explants (*n* = 4), (ii) sponge explants from the BCAA incubation experiment (*n* = 3) and the DIC incubation experiment (*n* = 3), (iii) and the 0.2 μm Sterivex filters (Merck Pty Ltd., Darmstadt, Germany) from the seawater control jars (*n* = 3) and sponge incubation jars (*n* = 2) from the BCAA and DIC incubation experiments was extracted using the DNeasy PowerSoil Pro Kit (QIAGEN, Hilden, Germany). Blank DNA extractions were included to account for possible handling contamination. The V4 region of the 16*S* rRNA gene was amplified and sequenced on an Illumina MiSeq (2 × 300 bp) at the Joint Microbiome Facility (JMF) of the Medical University of Vienna and the University of Vienna (JMF project IDs: JMF-2212-27, JMF-2303-04, and JMF-2407-25) using the primer pair 515F 5′-GTG YCA GCM GCC GCG GTA A-3′ (*76*) and 806R 5′-GGA CTA CNV GGG TWT CTA AT-3′ (*77*) (Biomers, Ulm, Germany), as previously described (*78*). Demultiplexing was performed with the Python package demultiplex (Laros JFJ, github.com/jfjlaros/demultiplex), allowing one mismatch for barcodes and two mismatches for linkers and primers. Amplicon sequence variants (ASVs) were inferred using the DADA2 R package v1.32 (*79*), applying the recommended workflow (https://f1000research.com/articles/5-1492). FASTQ reads 1 and 2 were trimmed at 220 and 150 nucleotides (nt), respectively, with allowed expected errors of 2. ASVs were classified using DADA2 against the SILVA database SSU Ref NR 99 release 138.1. ASVs classified as chloroplasts and mitochondria were removed from the ASV table. Singletons (ASVs that occur only once) were removed from the ASV table for the subsequent data analysis. The relative abundances of the ASVs originating from the three dominant *I. basta* symbionts, together with the other bacterial and archaeal ASVs were calculated and visualized in R (*80*) using the packages phyloseq (*81*), dplyr (*82*), and ggplot2 (*83*).

### ^13^C and ^15^N enrichment in bulk sponge tissue

The ^13^C and ^15^N enrichment in the bulk tissue of sponge explants from the DIC and BCAA incubation experiments and the natural abundance of these isotopes in field-control explants were measured by elemental analyzer–IRMS (EA-Isolink coupled via ConFlo IV interface to Delta V Advantage IRMS, Thermo Fisher Scientific, Bremen, Germany). Prior to analysis, the subsampled fragments of sponge explants (*n* = 9) were freeze-dried and ground to a fine powder in a Retsch MM200 ball mill (Retsch, Hanau, Germany) for 5 min. Samples for ^13^C enrichment measurements were decalcified with 0.4 M HCl overnight to remove inorganic carbon and dried at 65°C for 48 hours. All samples were subsequently weighed (between 0.5 and 1.0 mg) into tin capsules and stored dry until measured. The EA-IRMS system was calibrated for carbon and nitrogen content and isotope composition using laboratory standards (sugar-amino acid mixes) referenced against international standards (USGS40, USGS41a, and USGS64). Heavy stable isotope abundances of carbon and nitrogen were measured in untreated (natural abundance) samples and in isotopically enriched ones as atomic % ^13^C (at % ^13^C) and atomic % ^15^N (at % ^15^N), and the atomic % of treated samples were then contrasted against control samples by calculating the atomic percent enrichment (APE) of ^13^C and ^15^N.

### CARD-FISH–NanoSIMS of sponge tissue sections

Subsamples of sponge explants of both labeling experiments (BCAA and DIC) and of a natural isotope abundance control sample of an untreated *I. basta* individual were fixed in 4% paraformaldehyde (ProSciTech, Kirwan, Australia) overnight at 4°C, rinsed three times with ice-cold 1× phosphate-buffered saline (PBS), and subsequently stored in PBS:ethanol (1:1) at −20°C. Thin sections of sponge tissue were prepared at the Histology Facility of the Vienna Biocenter (Vienna, Austria). In brief, fixed samples were washed for 1 hour in 1× PBS and stored overnight at 4°C in 30% sucrose to preserve tissue morphology. Samples were subsequently transferred to 50% sucrose:Tissue-Tek OCT (optimal cutting temperature) compound embedding medium (Sakura, Torrance, USA) for embedding and stored overnight at 4°C. The embedded samples were transferred to isopentane and frozen in liquid nitrogen. Embedded tissue blocks were kept either on dry ice or at −80°C until 5 μm cryosections were prepared and transferred onto indium tin oxide (Sigma-Aldrich, Darmstadt, Germany) wafers.

Cryo-medium residues were removed by submerging the wafers for 3 min each in 50, 80, and 96% ethanol. Subsequently, samples were dried at room temperature. To enable correlative imaging of fluorescence visualization of the sponge symbionts via CARD-FISH and their single-cell isotope incorporation via NanoSIMS (50L, Cameca, Gennevilliers, France), regions of interest (ROIs) were marked using a laser microdissection microscope (Leica LMD 7000, Wetzlar, Germany).

CARD-FISH was performed following Pernthaler (*84*), using probes Arch915 (*85*), AlfD729 (*86*), and GamD1137 (*13*), together with newly designed helper probes (table S1; Biomers, Ulm, Germany), with some modifications. For all CARD-FISH experiments, negative controls using probe NonEUB (*87*) were also performed. Prior to CARD-FISH, sponge tissue sections were encircled using a pap-pen (Kisker, MKP-1, Steinfurt, Germany) to avoid loss of buffers during the CARD-FISH protocol. The samples were permeabilized using lysozyme (10 mg ml^−1^ in 0.05 M EDTA and 0.1 M tris-HCl, pH 8; 60 min at 37°C). After washing twice in deionized water, the samples were further permeabilized using HCl (0.2 M, 10 min, room temperature), again washed twice in deionized water, and subsequently dried. Endogenous peroxidases were inactivated using a freshly prepared 0.3% H_2_O_2_ solution in 1× PBS (30 min, room temperature), and samples were again washed in deionized water and dried. We performed two consecutive rounds of hybridization (each 3 hours, 46°C, at the respective optimal formamide concentration; see table S1) and signal amplification (each 30 min, 46°C), using Alexa488-labeled tyramides with probe Arch915 and Alexa594-labeled tyramides with probe AlfD729. Between each round of hybridization, the previously used probes were inactivated by incubation in 0.01 M HCl (10 min, at room temperature), followed by washing in deionized water twice.

Following CARD-FISH, samples were counterstained using DAPI (1 μg ml^−1^, 10 min, room temperature), washed twice in deionized water, and let dry, before embedding in CitiFluor AF1 (EMS, Morgantown, USA). CARD-FISH signals of the AOA symbiont *Ca.* N. ianthellae and the alphaproteobacterial symbiont *Ca.* L. ianthellae, as well as LMD markings, were visualized using a confocal laser scanning microscope (SP7, Leica, Wetzlar, Germany, equipped with a white light laser). Cells of the gammaproteobacterial symbiont *Ca.* T. ianthellae were identified based on DAPI signals showing their characteristic rod-shaped morphology, which was confirmed in separate CARD-FISH experiments using the specific probe GamD1137 (*13*). To capture symbionts within the entire volume of the sponge sections, *z*-stacks were recorded and projected into one plane (maximum projection algorithm). After visualization, the embedding medium was removed from the sections by washing in deionized water three times for 5 min.

NanoSIMS measurements were carried out using a NanoSIMS 50L instrument (Cameca, Gennevilliers, France) at the Large-Instrument Facility for Environmental and Isotope Mass Spectrometry, University of Vienna. Prior to data acquisition, analysis areas were preconditioned by rastering with a high-intensity, defocused Cs^+^ ion beam using the extreme low ion impact energies (EXLIE) deposition procedure reported previously (*88*): high energy (HE; 16 keV) at 100 pA beam current to a fluence of 5 × 10^14^ ions per cm^2^; EXLIE (50 eV) at 400 pA beam current to a fluence of 5 × 10^16^ ions per cm^2^; and HE to an additional fluence of 2.5 × 10^14^ ions per cm^2^. The multicollection detector assembly was configured to enable parallel detection of the following secondary ions: ^12^C^12^C^−^, ^12^C^13^C^−^, ^12^C^14^N^−^, ^12^C^15^N^−^, ^31^P^−^, and ^32^S^−^. The mass spectrometer was tuned to achieve a mass resolving power (MRP) >9000 (as defined by Cameca) to ensure accurate separation of C_2_^−^ and CN^−^ secondary ions. Data were acquired as multilayer image stacks by sequential rastering of a finely focused Cs^+^ primary ion beam (∼1 pA), achieving a lateral resolution of ∼80 nm. Scanning areas ranged from 25 × 25 to 50 × 50 μm^2^ at 512 × 512 pixel resolution, with a per-pixel dwell time of 1.5 ms per pixel per cycle. Between 38 and 47 cycles were acquired per scanning area.

NanoSIMS images were processed using Look@NanoSIMS (version 24-12-20) implemented in MATLAB R2024b (academic license). Image stacks (cycles) were aligned to correct for positional drift due to room temperature fluctuations, primary beam instability, or stage movement. Secondary ion intensities were corrected on a per-pixel basis for detector dead time and quasi-simultaneous arrival effects using sensitivity correction factors of 1.06 for C_2_^−^, 1.05 for CN^−^, and 1.10 for all other ions. All measurement cycles were accumulated, and ROIs, corresponding to individual microbial cells or sponge nuclei, were manually defined based on phosphorus ion distribution maps in correlation with CARD-FISH and DAPI signals. Because ROIs were defined using all accumulated cycles per measurement area, it is possible that some ROI enrichments are under- or overestimated when secondary ions from tissue below or above microbial cells were ablated. We did not take any dilution effects from the CARD-FISH procedure into account for our calculations, as they differ depending on the CARD-FISH protocol used, cell types, and growth stages (*89*–*91*), which cannot be constrained in environmental samples. As such, the reported growth rates, doubling times, and amounts of freshly fixed carbon and nitrogen are considered conservative.

Carbon isotope composition (expressed as at % ^13^C) was calculated from C_2_^−^ secondary ion images (Eq. 1)

C13 at %=C12C−13(2∗C12C−12+C12C−13)×100 (1)

Similarly, nitrogen isotope composition (at % ^15^N) was derived from CN^−^ secondary ion images (Eq. 2)

N15 at %=C12N−15(C12N−14+C12N−15)×100 (2)

Only ROIs with an associated Poisson error of <5% (for carbon or nitrogen isotopic composition, respectively) were considered for further calculations and used for visualization.

Single-cell carbon-based growth rates (GR) were calculated (Eq. 3) following Martínez-Pérez *et al.* (*92*)

GR (day−1)=log2[mediumC13 at %excess/(mediumC13 at %excess−cellC13 at %excess)]time (days) (3)

where time is the incubation time in days. Medium ^13^C at %_excess_ was obtained for DIC incubations (Eq. 4), using the measured ^13^C-DIC concentration at the beginning of the incubation

mediumC13 at %excess (DIC)=mediumC13 at %DIC−mediumC13 at %natural abundance (4)

Medium ^13^C at %_excess_ for BCAA incubations was determined (Eq. 5), using the measured ^15^N^13^C BCAA concentrations at the beginning of the incubation

mediumC13 at %excess (BCAA)=N15C13 BCAA (μmol)pore water BCAA (μmol)+N15C13 BCAA (μmol)×100 (5)

Cell ^13^C at %_excess_ was obtained by subtracting the mean ^13^C at % of all single symbiont cells in natural abundance sponge sections (1.06 at % ^13^C) from single-cell ^13^C at % values determined in ^13^C-DIC and ^15^N^13^C BCAA incubated samples.

Doubling times (DT) were inferred from (Eq. 6)

DT (days)=1/GR (6)

The amount of newly assimilated carbon (and analogously for nitrogen) from BCAA was estimated (Eq. 7) following Krupke *et al.* (*93*)

C uptake (fmol C cell−1day−1)=(cellC13 at%excessmediumC13 at %excess)×cellular carbon (fmol C cell−1)time (days) (7)

Cellular carbon contents were estimated following Khachikyan *et al.* (*94*), using ROI dimensions from NanoSIMS data (Eqs. 8 and 9)

$$\text{cellular carbon}\left(\text{fg}\;C\;\text{cel}l^{-1}\right)=197\times \text{cell volume}\;{\left(\mu m^{3}\right)}^{0.46}$$ (8)

$$\text{cellular carbon}\;\left(\text{fmol}\;C\;\text{cel}l^{-1}\right)=\frac{\text{cellular carbon}\left(\text{fg}\;C\;\text{cel}l^{-1}\right)}{12\;\left(\text{fg}\;{\text{fmol}}^{-1}\right)}$$ (9)

where cell volume (in cubic micrometers) was estimated from NanoSIMS ROI dimensions, assuming prolate sphere shapes (Eq. 10) (*95*)

$$\text{cell volume}\;\left(\mu m^{3}\right)=\left(\frac{\pi }{6}\right)\times \left[\text{cell width}\;{\left(\mu m\right)}^{2}\right]\times \text{cell length}\;\left(\mu m\right)$$ (10)

Cellular nitrogen content was estimated using the Redfield ratio of C:N = 6.625 (Eq. 11)

$$\text{cellular nitrogen}\;\left(\text{fmol}\;N\;\text{cel}l^{-1}\right)=\frac{\text{cellular carbon}\;\left(\text{fmol}\;C\;\text{cel}l^{-1}\right)}{6.625}$$ (11)

We assumed that the sponge symbionts are exposed to the concentrations of experimentally added isotope tracers inside the sponge holobiont, i.e., that the DIC concentrations and ^13^C-isotope enrichment were identical in the incubation seawater (medium) and inside the sponge. For BCAA, we assumed that the experimentally added ^13^C^15^N-BCAA was available to the sponge symbionts at equal concentrations as supplied to the incubation seawater. Furthermore, we assumed that the sponge symbionts are additionally exposed to native BCAA concentrations of natural isotope abundance measured in sponge pore water. Whether these assumptions hold true requires additional research; however, we assume that if the sponge holobiont indeed reduces the availability of stable isotope-labeled compounds for its symbionts by providing an uptake barrier, this effect would be similar for both BCAA and DIC.

## Supplementary Materials

### The PDF file includes:

Figs. S1 to S10 Table S1 Legends for data S1 to S7 References
