# Supplementary material for: Branched-chain amino acid assimilation enables mixotrophy of ammonia-oxidizing archaeal sponge symbionts
Source: Sci Adv. 2026 Sep 4;12(36):eaef9450. doi: 10.1126/sciadv.aef9450 (PMC13552627; doi:10.1126/sciadv.aef9450)
Supplement: Supplementary file 1 [file sciadv.aef9450_sm.pdf]

Science Advances
AAAS

Supplementary Materials for

Branched-chain amino acid assimilation enables mixotrophy of ammonia-oxidizing archaeal sponge symbionts

Bettina Glasl *et al.*

Corresponding author: Bettina Glasl, bettina.glasl@univie.ac.at;
Katharina Kitzinger, katharina.kitzinger@univie.ac.at

Sci. Adv. **12**, eaef9450 (2026)
DOI: 10.1126/sciadv.aef9450

The PDF file includes:

Figs. S1 to S10
Table S1
Legends for data S1 to S7
References

Other Supplementary Material for this manuscript includes the following:

Data S1 to S7

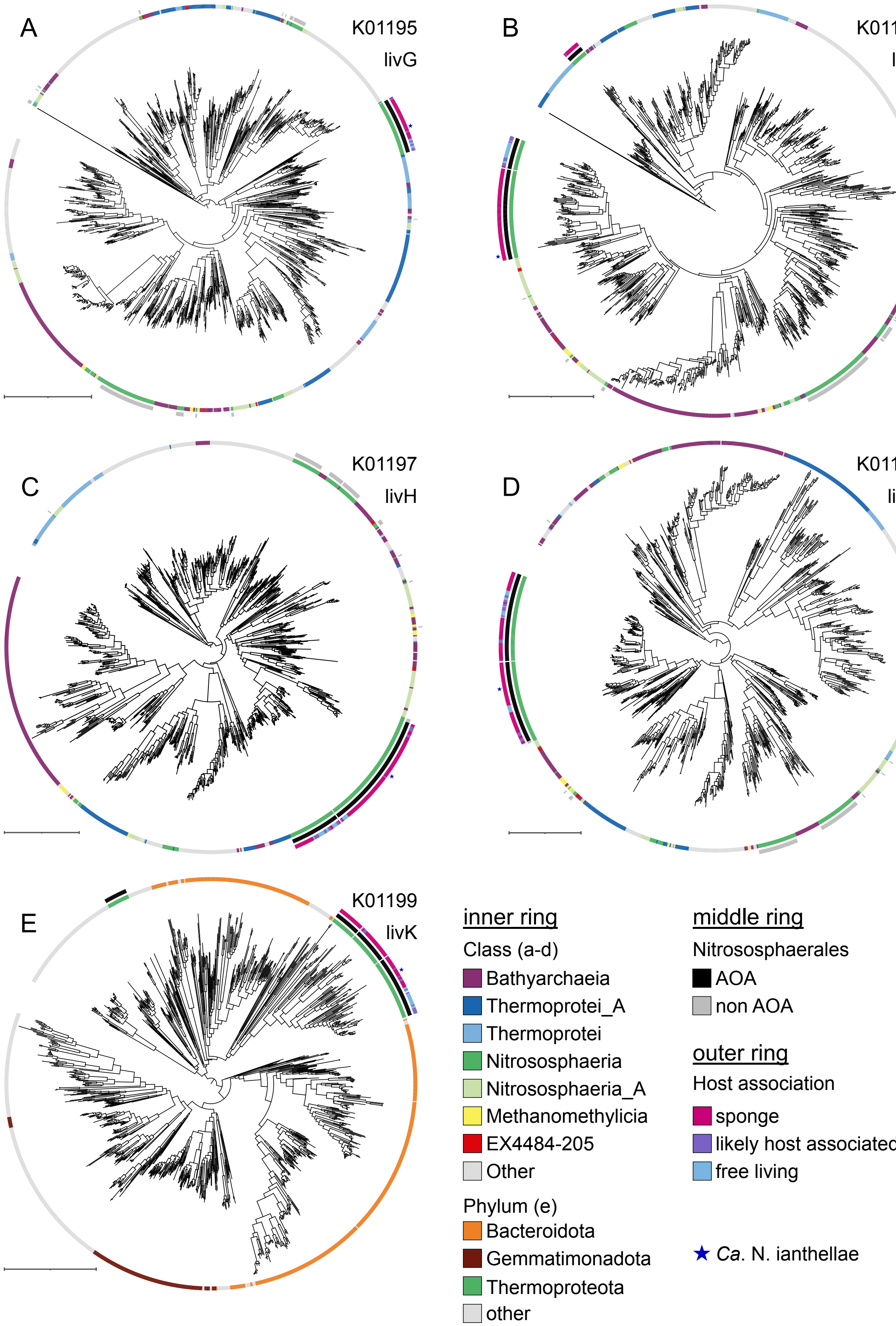

A
K01195
livG
B
K01196
livF
C
K01197
livH
D
K01198
livM
E
K01199
livK
inner ring
Class (a-d)
Bathyarchaeia
Thermoprotei_A
Thermoprotei
Nitrososphaeria
Nitrososphaeria_A
Methanomethylicia
EX4484-205
Other
Phylum (e)
Bacteroidota
Gemmatimonadota
Thermoproteota
other
middle ring
Nitrososphaerales
AOA
non AOA
outer ring
Host association
sponge
likely host associated
free living
★ Ca. N. ianthellae

Fig. S1. Amino acid phylogenies of the phylogenetic context of the branched-chain amino acid (BCAA) transporter subunits in ammonia-oxidizing archaea (AOA). The taxa included in the phylogenies were chosen based on a phylogenetic analysis of the full datasets of each subunit (see *Materials and Methods*). The phylogenetic neighborhood of the ATP-binding subunits *livG* (**A**) and *livF* (**B**), and the transmembrane subunits *livH* (**C**) and *livM* (**D**), largely consists of conserved taxa within the archaeal *Thermoproteota* phylum that also includes the *Nitrososphaerales*. While this suggests shared ancestry, the topology of the four phylogenies is inconsistent, making it impossible to determine the specific donor lineage from these analyses. In contrast, the phylogenetic neighborhood of the AOA substrate binding subunit *livK* (**E**) consists primarily of sequences of the bacterial phylum *Bacteroidota*, indicating a different ancestry than the rest of the complex. Inner colored rings around the phylogenies depict taxonomic affiliation at the class (**A-D**) or phylum (**E**) level. The middle colored ring indicates sequences from genomes in the *Nitrososphaerales* order, with AOA in black and other *Nitrososphaerales* in grey. The outer colored ring indicates whether the source genome of a sequence is host-associated based on the source data of the genome and the metagenomes in which it is detected. The position of the *Candidatus* Nitrosospongia ianthellae sequences is indicated with a blue star. The scale bar at the bottom right of each phylogeny indicates one substitution per site.

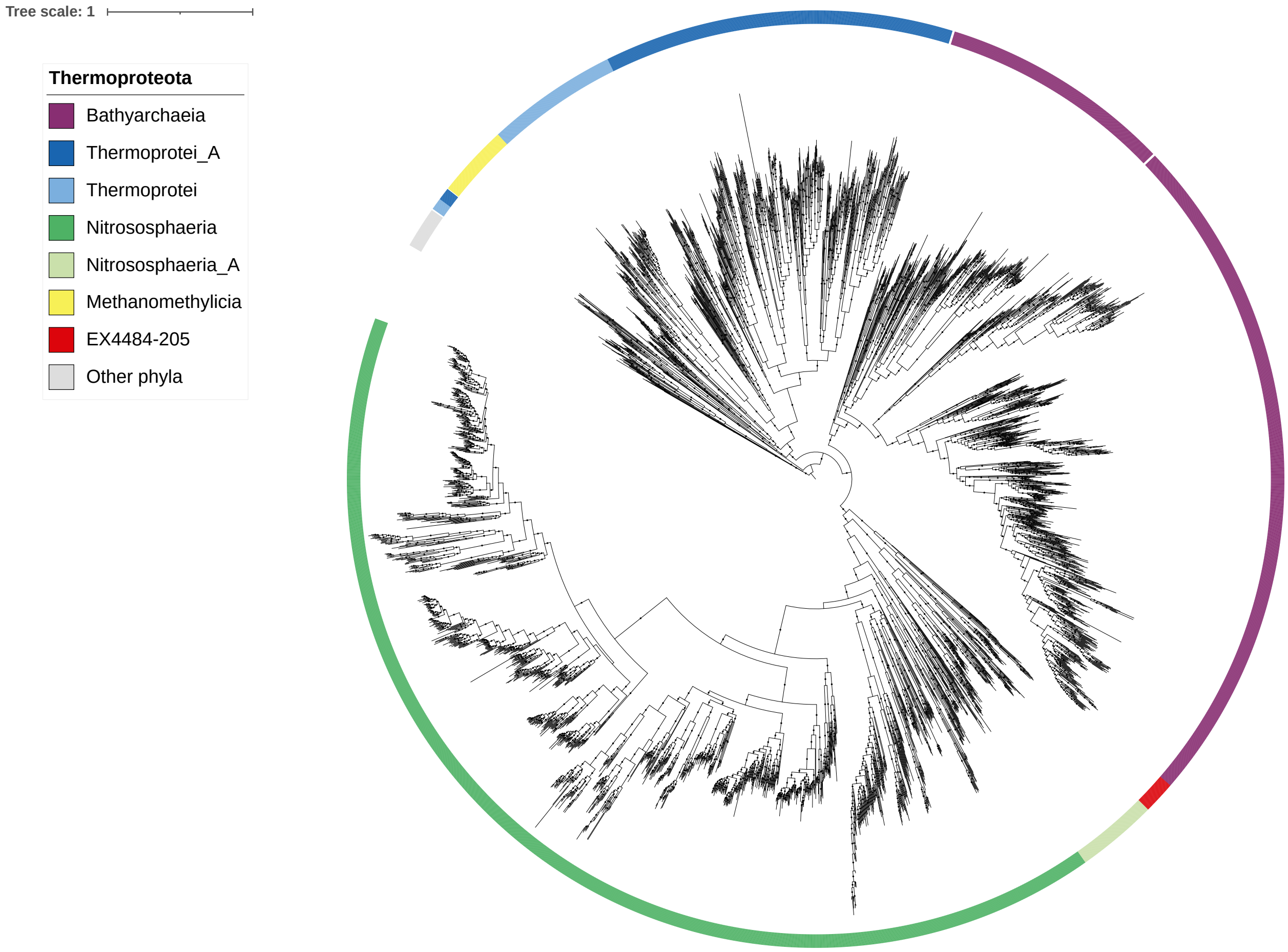

Fig. S2. *Thermoproteota* genome tree. The concatenated marker gene phylogeny of 3,584 genomes in the *Thermoproteota* phylum is based on 76 conserved archaeal marker genes. The classes within the *Thermoproteota* phylum are indicated using the colorstrip on the outer edge of the tree. Black circles on the branches indicate >=95% bootstrap support. The tree is rooted using *Korachaeota* genomes as the outgroup. At the base of the *Thermoproteota* phylum, this phylogeny does not agree with the taxonomic assignment, but this has no bearing on the *Nitrososphaeria* discussed in this study.

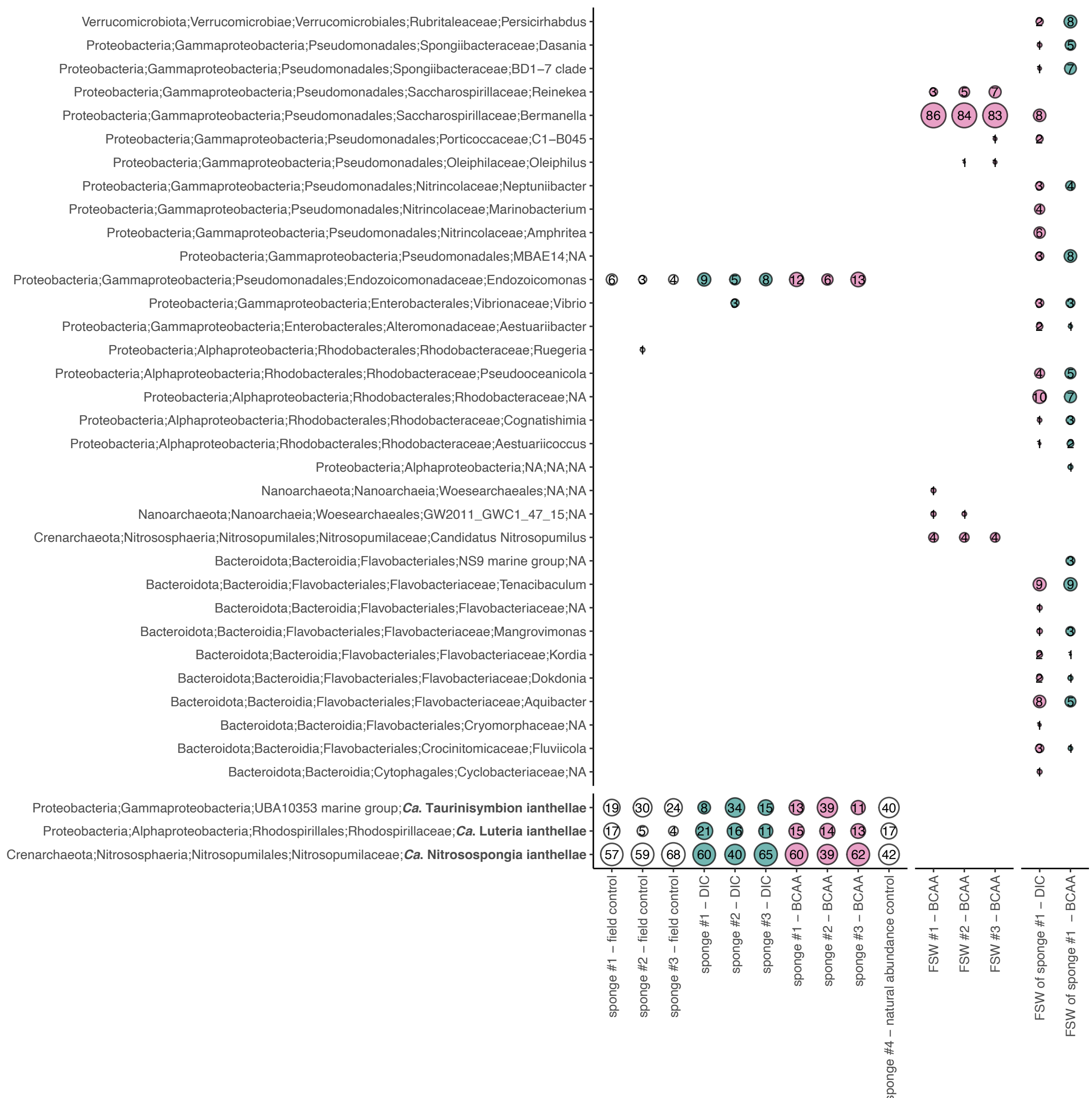

Fig. S3. Microbial community composition of *Ianthella basta* and seawater samples. The relative abundance of bacterial and archaeal microbiome members in *I. basta* individuals was determined using 16S rRNA gene amplicon sequencing. The microbiome composition of the sponge explants remained stable throughout the stable isotope incubation experiments (DIC = ^{13}C-labeled dissolved inorganic carbon incubation experiment, BCAA = ^{13}C^{15}N-labeled branched-chain amino acid incubation experiment). Sponge-explants were subsampled prior to the experiments (field control) and at the end of the 24h incubation experiments. The microbial community associated with the filtered seawater control samples, collected at the end of the 24h incubation period, of the ^{13}C^{15}N-labeled BCAA incubation experiment (FSW BCAA, pore size for filtration 0.22 mm) and the incubation water of the sponge incubations with labeled DIC and BCAA (FSW of sponge #1; fig. S6) varied substantially from the *I. basta* microbiome. In the filtered seawater BCAA control jars, we observed growth of putative heterotrophs belonging to the genus *Bermanella* (*97*), which either passed through the filter pores (0.22 µm) or were present

in the incubation jars/room during experimental setup, which was done under non-sterile conditions. The growth of these putative heterotrophs can also explain the consumption of BCAA in the filtered seawater incubation without sponge explants (fig. S5). The relative abundances of the three dominant *I. basta* symbionts are displayed at the amplicon sequence variant (ASV) level; the relative abundances of the other microbiome ASVs are summarized at the family level.

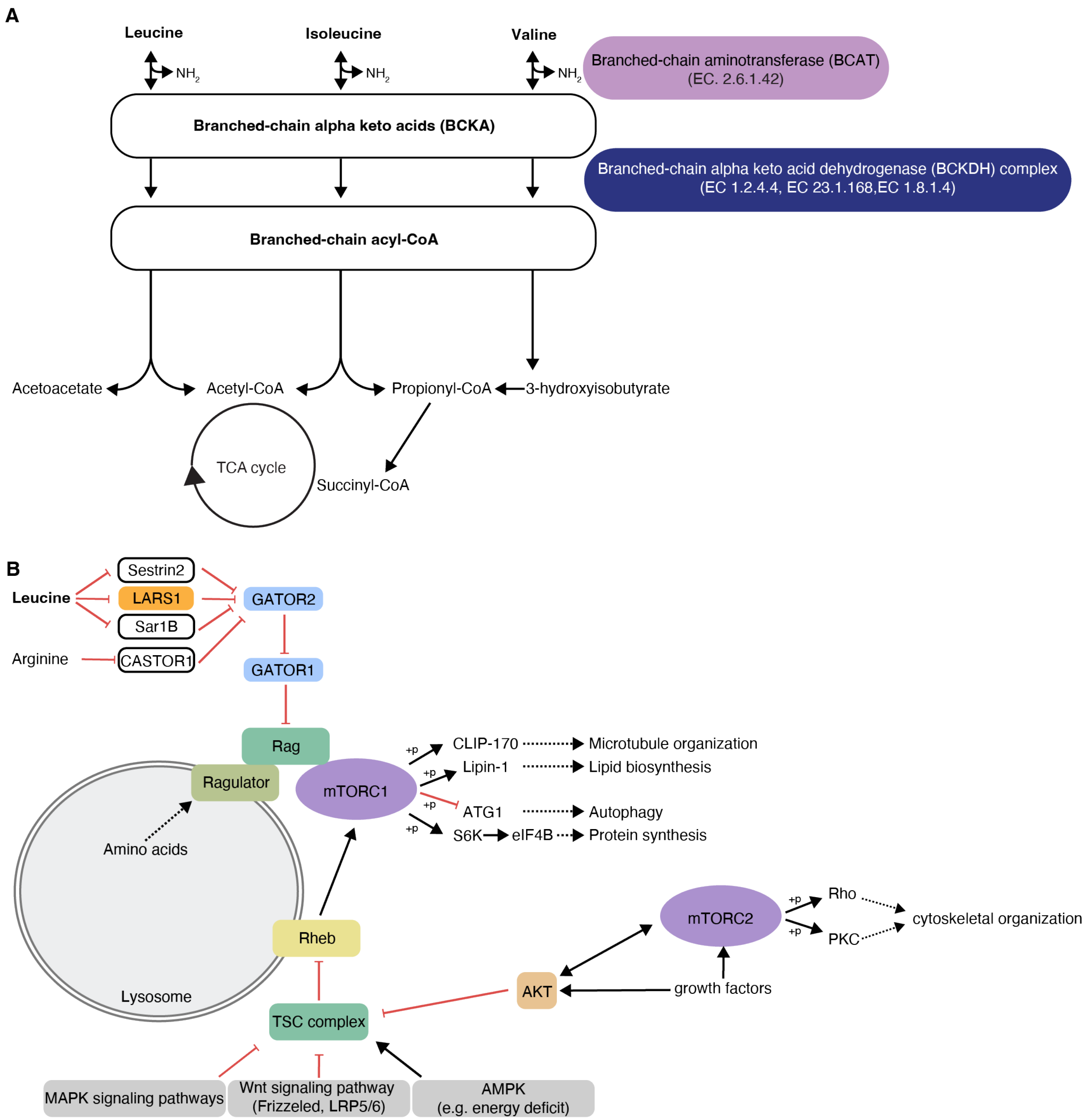

Fig. S4. Genomic and transcriptomic capacity of *Ianthella basta* to utilize branched-chain amino acids (BCAA). (**A**) The *I. basta* (GCA_964019415.1) sponge genome encodes and transcribes for the complete branched-chain amino acid (BCAA) degradation pathway. The first step is the reversible transamination of BCAA to its respective branched-chain alpha keto acids (BCKA). The second step is the oxidative decarboxylation of the BCKA to branched-chain acyl-CoA derivatives, which is catalyzed by the branched-chain alpha-keto acid dehydrogenase (BCKDH) complex. Succinyl-CoA and acetyl-CoA are the end products of the valine, leucine, and isoleucine catabolism. Succinyl-CoA and acetyl-CoA serve as substrates for the tricarboxylic acid (TCA) cycle. (**B**) BCAA, especially leucine, are key regulators of the mechanistic target of rapamycin complex 1 (mTORC1), a central protein complex of eukaryotic cells. Leucine can modulate the mTORC1 complex and thus stimulate microtubule organization, lipid biosynthesis,

and protein synthesis, but inhibits autophagy. The *I. basta* genome encodes and transcribes all genes of the mTORC1 complex including the leucine sensing protein LARS1 (leucyl-transfer RNA (tRNA) synthetase 1; K01889). It lacks homologous genes encoding for other leucine sensing proteins such as Sestrin2 and Sar1B, and the arginine sensing protein CASTOR1. KEGG Orthology (KO) assignment and KEGG pathway reconstruction were performed using GhostKOALA (*72*). The pathway maps were generated with KEGG mapper and modified in Adobe Illustrator.

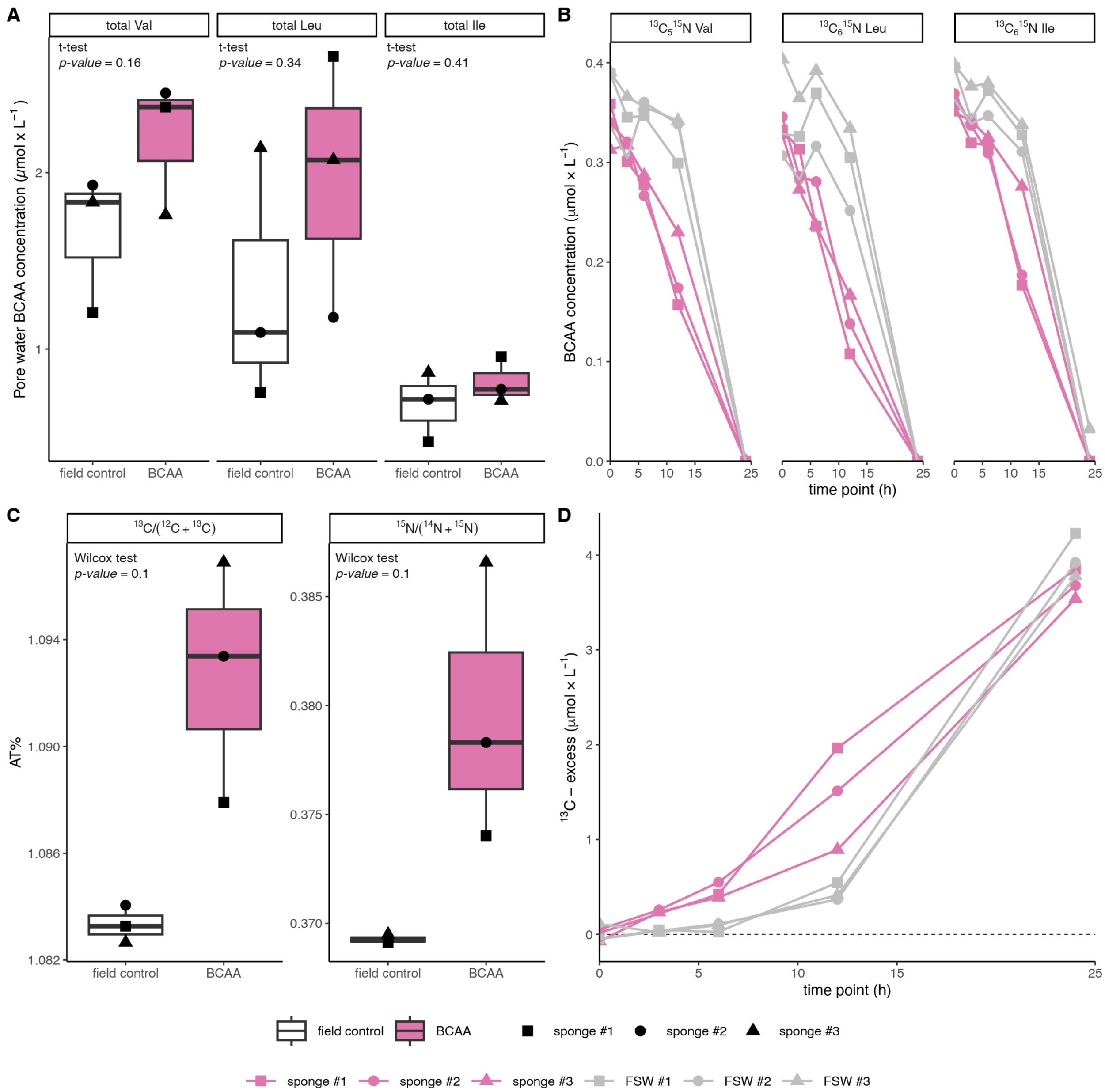

Fig. S5. Bulk BCAA concentration in and turnover by the *Ianthella basta* holobiont. (**A**) Total branched-chain amino acid (BCAA) concentrations (i.e., valine, leucine, and isoleucine) in readily extractable pore water from *I. basta* holobiont samples (field control = sponge pore water samples collected prior to incubation, BCAA = sponge pore water samples collected after 24h BCAA incubation experiment). Boxplots depict the 25–75% quantile range, with the centre line depicting the median (50% quantile); whiskers encompass data points within 1.5× the interquartile range. (**B**) Depletion of isotopically labeled BCAA in the incubation water over the 24h incubation period. Consumption of BCAA was faster over the first 12h of the experiment in incubations containing sponge explants (0.04 ± 0.004 (SE) µmol L^{-1} h^{-1} , p-value = 0.001) compared to filtered seawater (non-significant decrease 0.01 ± 0.01 (SE) µmol L^{-1} h^{-1}, p-value = 0.059). (**C**) ^{13}C and ^{15}N-enrichment in *I. basta* holobiont biomass samples (field control = sponge biomass samples collected prior to incubation, BCAA = sponge biomass samples collected after 24h BCAA incubation experiment). Boxplots depict the 25–75% quantile range, with the centre

line depicting the median (50% quantile); whiskers encompass data points within 1.5× the interquartile range. Note, the field control *vs*. BCAA treatment showed complete rank separation ($W = 0$), corresponding to a rank-biserial effect size of $r = 1.0$, indicating a very strong difference despite the limited sample size ($n = 3$ per treatment). The exact Wilcoxon p-value was 0.1, which reflects low statistical power rather than absence of effect. (**D**) The respiration of isotopically labeled BCAA results in an excess of ^{13}C-labeled CO_2 (dissolved inorganic carbon = DIC) in the incubation water over the course of the 24h experiment. Note that also in incubations containing only filtered seawater (no sponge explants), added BCAA are depleted over the course of the incubation, and largely respired to CO_2. This can be explained by the presence and growth of some heterotrophic cells that are capable of respiring BCAA, which either passed through the filter pores (0.22 µm) or were present in the incubation jars/room during experimental setup, which was done under non-sterile conditions. Putative heterotrophs growing during incubation belong to the genus *Bermanella* (*97*), which we detected differentially enriched in filtered seawater incubations without sponge explants (see fig. S3).

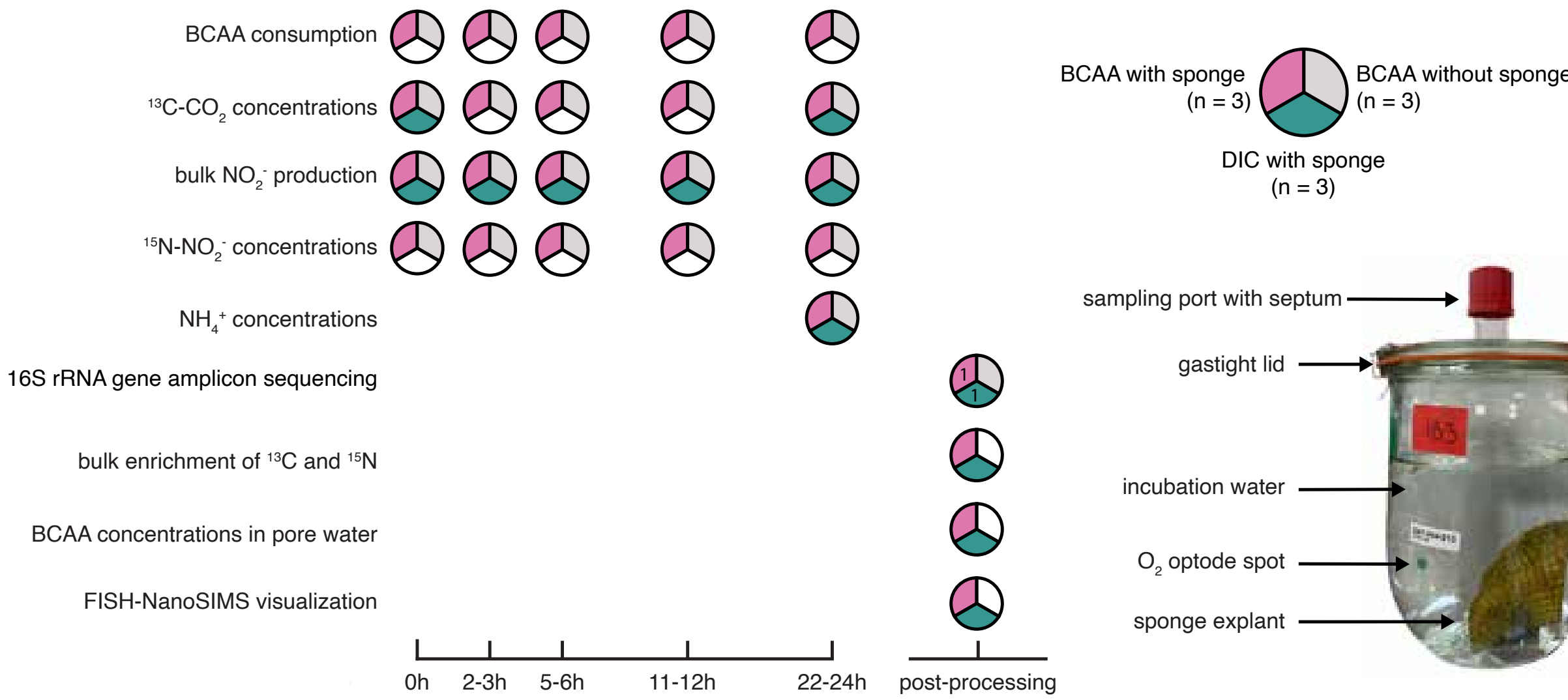

Fig. S6. Sampling scheme and experimental setup for the stable isotope incubation experiments. The incubation jars were filled with filtered seawater and amended with a $^{15}N^{13}C$-labeled branched-chain amino acid (BCAA) mix containing equimolar concentrations of valine, leucine, and isoleucine, or with ^{13}C-dissolved inorganic carbon (DIC). Incubation water samples for BCAA consumption, ^{13}C-CO_2 concentrations, bulk NO_2^-, ^{15}N-NO_2^-, and NH_4^+ concentrations were collected at regular intervals via the sampling port throughout the incubation period (up to 24 hours). At the end of the incubation experiments, the sponge explants were subsampled for (i) 16S rRNA gene amplicon sequencing, (ii) measurement of bulk enrichment of ^{13}C and ^{15}N in the sponge tissue, (iii) extraction of pore water samples, and (iv) fixation for FISH-NanoSIMS. Incubation jars remained closed throughout the incubation period to avoid exchange of the headspace with the atmosphere.

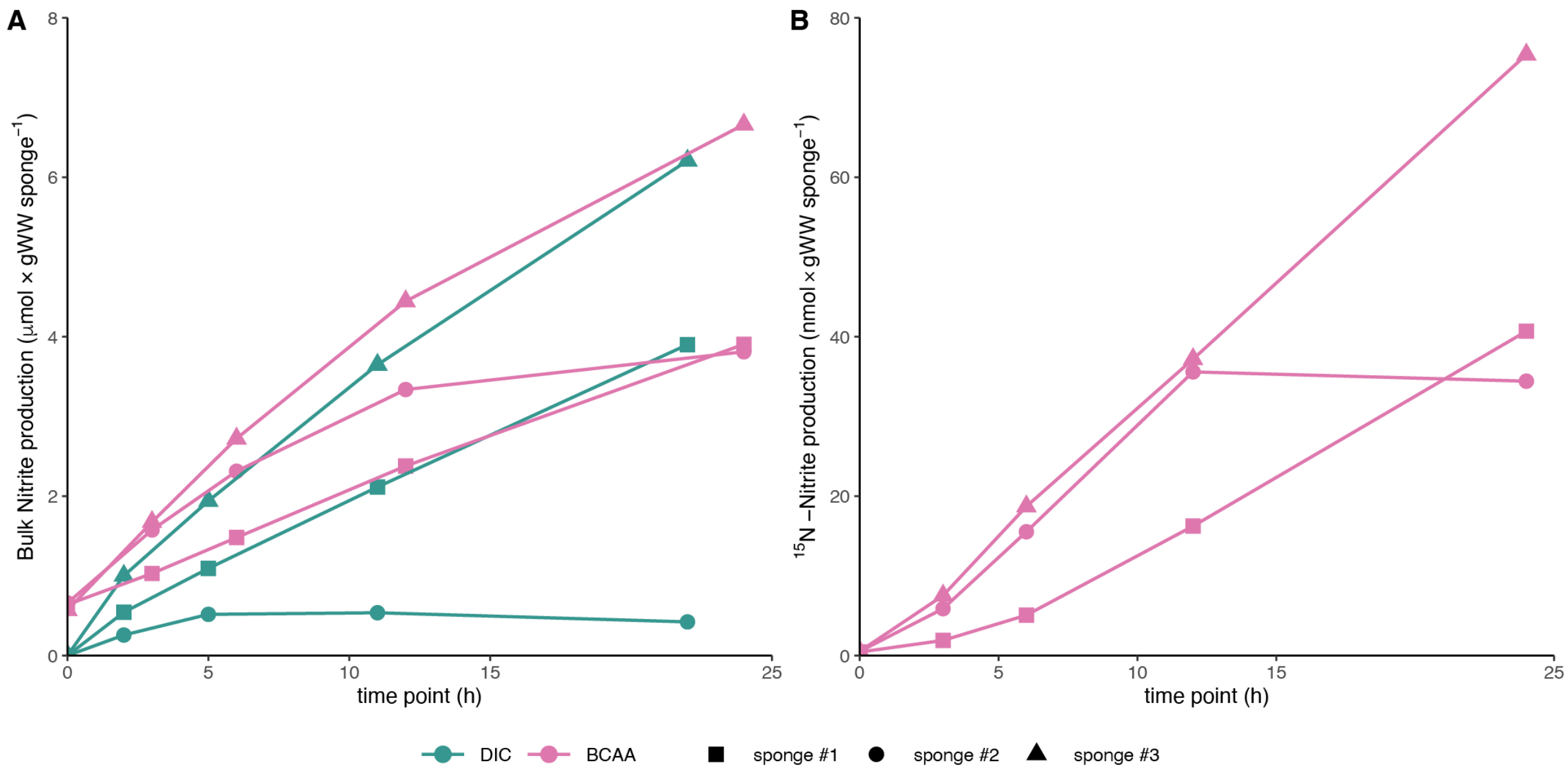

Fig. S7. Nitrification activity of the *Ianthella basta* sponge holobiont. (**A**) Bulk nitrite (NO_2^-) production per sponge individual during DIC and BCAA incubation experiments. Nitrite formation in each incubation jar was normalized to the wet weight (ww) of the sponge explants. (**B**) ^{15}N-nitrite (NO_2^-) production per sponge individual during the BCAA incubation experiment. The formation of ^{15}N-nitrite was normalized to the wet weight (ww) of the sponge explants in each incubation jar. Bulk and ^{15}N-nitrite production rates of sponge and seawater control jars (in µmol per L) are shown in Fig. 3.

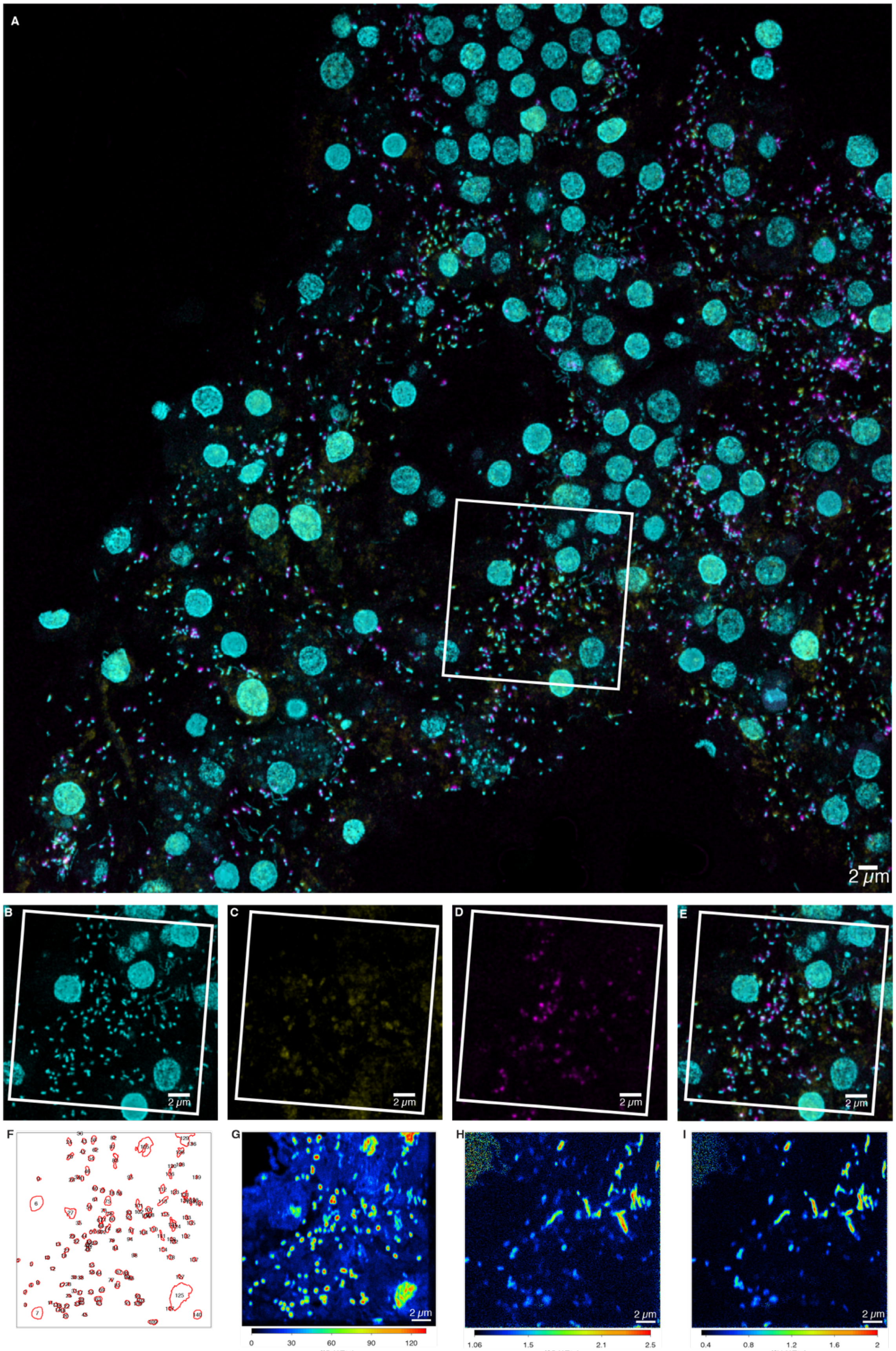
A
2 μm
B
2 μm
C
2 μm
D
2 μm
E
2 μm
F
G
2 μm
0 30 60 90 120
31P (AT%)
H
2 μm
1.06 1.5 2.1 2.5
13C (AT%)
I
2 μm
0.4 0.8 1.2 1.6 2
15N (AT%)

Fig. S8. Fluorescence and NanoSIMS images from the branched-chain amino acid (BCAA) incubation experiment. Correlative imaging showing CARD-FISH and NanoSIMS images sequential obtained from the same cryosection position; thus, all panels show the identical cells (the same cryosection position is also shown in Fig. 4). (**A**) Overlay of CARD-FISH images and DAPI showing the ammonia-oxidizing archaeal symbiont *Ca*. Nitrosospongia ianthellae (magenta, stained by probe Arch915), the alphaproteobacterial symbiont *Ca*. Luteria ianthellae (yellow, stained by probe AlfD729), the gammaproteobacterial symbiont *Ca*. Taurinisymbiont ianthellae (cyan, counterstained with DAPI), and sponge nuclei (nuc, cyan, counterstained with DAPI) in a 5 µm cryosection of the *Ianthella basta* holobiont. The white quadrat (25 x 25 µm) highlights the region of interest selected for NanoSIMS imaging. Zoomed-in visualization of (**B**) DAPI signals, (**C**) CARD-FISH signals of the ammonia-oxidizing archaeal symbiont *Ca*. Nitrosospongia ianthellae, (**D**) CARD-FISH signals of the alphaproteobacterial symbiont *Ca*. Luteria ianthellae, and (**E**) the overlay of the three individual channels. NanoSIMS images including (**F**) the defined regions of interest (ROI) and the (**G**) ^{31}P-content, (**H**) ^{13}C-enrichment, and (**I**) ^{15}N-enrichment of measured ROIs in atomic percent (AT%).

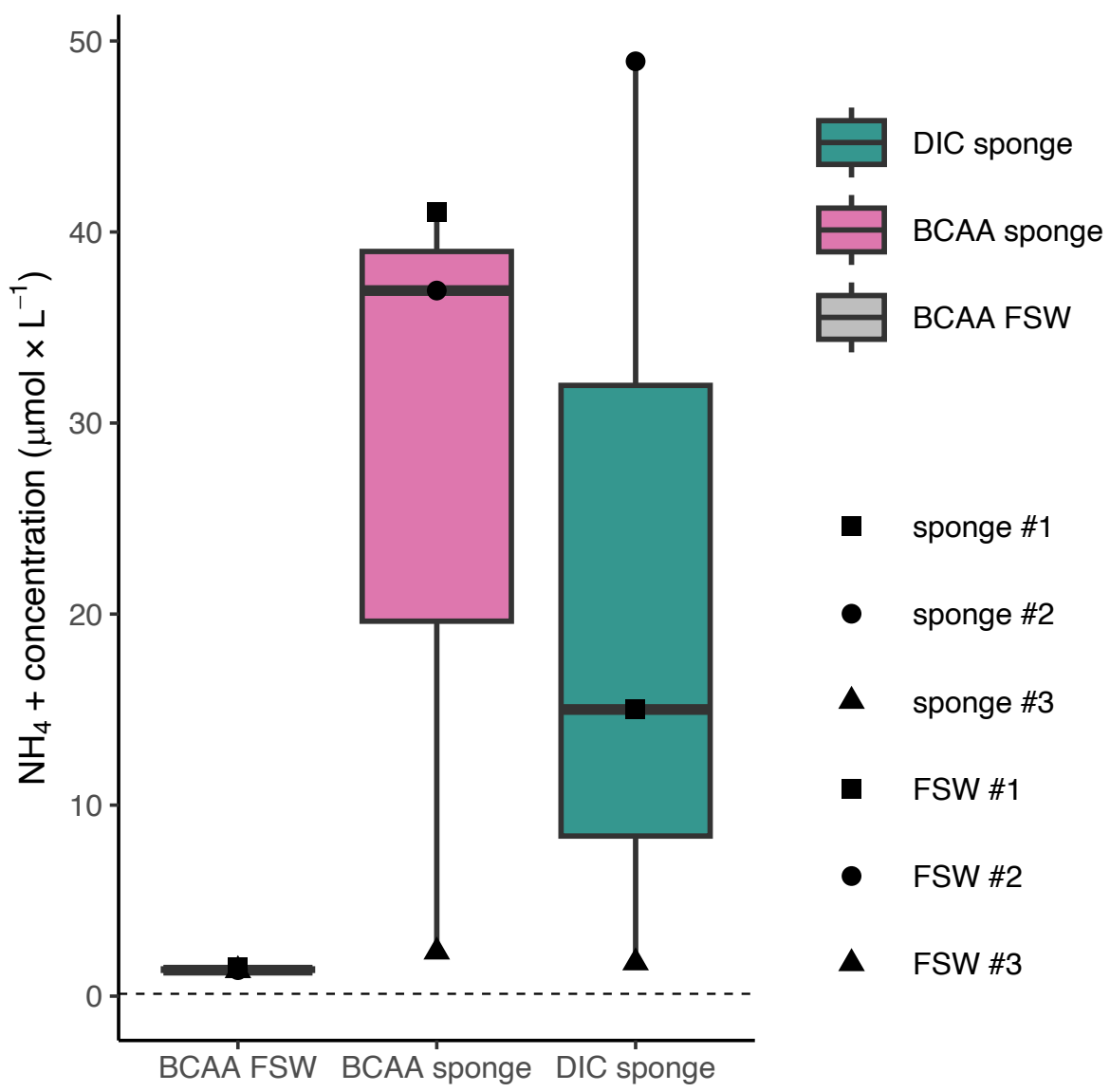

Fig. S9. Ammonium concentration in the stable isotope incubation experiments. Ammonium (NH_4^+) concentration in the seawater control jars filled with sterile filtered seawater (FSW) and in the sponge jars in the branched-chain amino acid (BCAA) incubation and dissolved inorganic carbon (DIC) experiments was measured at the end of the incubation experiments. Boxplots depict the 25–75% quantile range, with the centre line depicting the median (50% quantile); whiskers encompass data points within 1.5× the interquartile range. The dashed line represents the *in situ* seawater NH_4^+ concentration of the field sampling site.

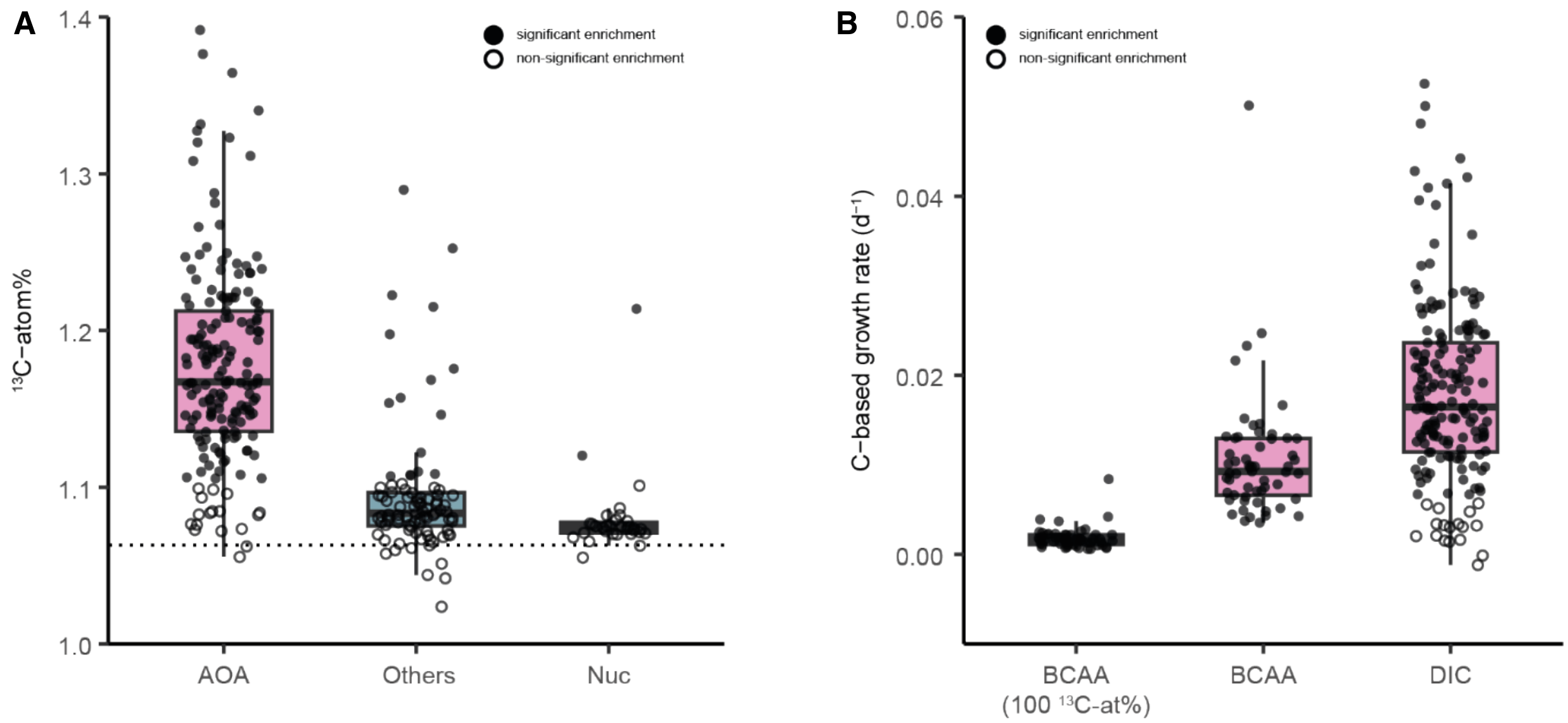

Fig. S10. Single cell ^{13}C-enrichment from ^{13}C-DIC and comparison of C-based AOA growth rates in incubations with ^{13}C^{15}N-BCAA and ^{13}C-DIC. (**A**) Single cell ^{13}C-enrichment from ^{13}C-DIC of AOA (*Ca.* N. ianthellae) and other microbial symbionts of *I. basta* (Others) and sponge nuclei (Nuc). Dotted line indicates natural ^{13}C abundance (in at%^{13}C) as measured for symbiont cells in non-incubated sponge biomass (1.06 at%^{13}C). AOA are significantly more enriched compared to the other microbial symbionts (one-sided Mann–Whitney U test, $W = 14{,}007$, $p < 2.2 \times 10^{-16}$). (**B**) Carbon-based growth rates of single AOA cells from ^{13}C^{15}N-BCAA, assuming that symbionts were either exposed to only fully labeled ^{13}C^{15}N-BCAA (BCAA (100 at%^{13}C)), or that symbionts were exposed to experimentally added ^{13}C^{15}N-BCAA and native BCAA at natural ^{13}C abundance present in the sponge pore water (BCAA). Growth rates inferred from ^{13}C-DIC assimilation are also depicted. Boxplots depict the 25–75% quantile range, with the centre line depicting the median (50% quantile); whiskers encompass data points within 1.5× the interquartile range. Open circles indicate non-significantly enriched cells.

Table S1. Oligonucleotide probes used in this study.

Oligo ID	target organism	Formamide concentration	Sequence	Helper Sequence (designed in this study)
Arch915 (*71*)	*Ca*. Nitrosospongia ianthellae	30%	GTG CTC CCC CGC CAA TTC CT	-
AlfD729 (*13*)	*Ca*. Luteria ianthellae	25%	CGG ACC TGG CGG CCG CTT	GCT ACT GGT GTT CTT CCC AAT CTC TAC GAA TTC CAC; CAC GCT TTC GCG CCT CAG CGT CAG T; CTG GGA GTT CCG CCG TCA TTT TCC G
GamD1137 (*13*)	*Ca*. Taurinisymbion ianthellae	25%	CTC AAA GTC CCC GCC ATT	GCG CTG GCA ACT AAG GAC AAG; CCG GTT TGT CAC CGG CAG TC
NonEUB (*87*)	non-sense probe	30%	ACT CCT ACG GGA GGC AGC	-

Data S1. (separate file)
Phylogenetic tree file, related to Fig. 1.

Data S2. (separate file)
Phylogenetic tree annotations, related to Fig. 1.

Data S3. (separate file)
Functional annotation of *Ca*. Nitrosospongia ianthellae metagenome assembled genome, related to Fig. 2.

Data S4. (separate file)
Bulk nitrification data, related to Fig. 3 and fig. S7.

Data S5. (separate file)
Source data for fig. S5. Bulk BCAA concentration in and turnover by the *Ianthella basta* holobiont.

Data S6 (separate file)
Source data for fig. S9. Ammonium concentration in the stable isotope incubation experiments.

Data S7 (separate file)
Source data for fig. S10. Single cell ^{13}C-enrichment from ^{13}C-DIC and comparison of C-based AOA growth rates in incubations with ^{13}C^{15}N-BCAA and ^{13}C-DIC.

REFERENCES

1. D. T. Schultz, S. H. D. Haddock, J. V. Bredeson, R. E. Green, O. Simakov, D. S. Rokhsar, Ancient gene linkages support ctenophores as sister to other animals. *Nature* **618**, 110–117 (2023).

2. C. Nielsen, Early animal evolution: A morphologist's view. *R. Soc. Open Sci.* **6**, 190638 (2019).

3. R. W. Van Soest, N. Boury-Esnault, J. Vacelet, M. Dohrmann, D. Erpenbeck, N. J. De Voogd, N. Santodomingo, B. Vanhoorne, M. Kelly, J. N. Hooper, Global diversity of sponges (Porifera). *PLOS ONE* **7**, e35105 (2012).

4. J. M. De Goeij, D. van Oevelen, M. J. A. Vermeij, R. Osinga, J. J. Middelburg, A. F. P. M. de Goeij, W. Admiraal, Surviving in a marine desert: The sponge loop retains resources within coral reefs. *Science* **342**, 108–110 (2013).

5. T. Thomas, L. Moitinho-Silva, M. Lurgi, J. R. Bjork, C. Easson, C. Astudillo-Garcia, J. B. Olson, P. M. Erwin, S. Lopez-Legentil, H. Luter, A. Chaves-Fonnegra, R. Costa, P. J. Schupp, L. Steindler, D. Erpenbeck, J. Gilbert, R. Knight, G. Ackermann, J. Victor Lopez, M. W. Taylor, R. W. Thacker, J. M. Montoya, U. Hentschel, N. S. Webster, Diversity, structure and convergent evolution of the global sponge microbiome. *Nat. Commun.* **7**, 11870 (2016).

6. U. Hentschel, J. Piel, S. M. Degnan, M. W. Taylor, Genomic insights into the marine sponge microbiome. *Nat. Rev. Microbiol.* **10**, 641–654 (2012).

7. N. S. Webster, T. Thomas, The sponge hologenome. *mBio* **7**, e00135–e00116 (2016).

8. M. W. Taylor, R. Radax, D. Steger, M. Wagner, Sponge-associated microorganisms: Evolution, ecology, and biotechnological potential. *Microbiol. Mol. Biol. Rev.* **71**, 295–347 (2007).

9. M. Haber, I. Burgsdorf, K. M. Handley, M. Rubin-Blum, L. Steindler, Genomic insights into the lifestyles of Thaumarchaeota inside sponges. *Front. Microbiol.* **11**, 622824 (2020).

10. S. J. Robbins, W. Song, J. P. Engelberts, B. Glasl, B. M. Slaby, J. Boyd, E. Marangon, E. S. Botte, P. Laffy, T. Thomas, N. S. Webster, A genomic view of the microbiome of coral reef demosponges. *ISME J.* **15**, 1641–1654 (2021).

11. D. Steger, P. Ettinger-Epstein, S. Whalan, U. Hentschel, R. de Nys, M. Wagner, M. W. Taylor, Diversity and mode of transmission of ammonia-oxidizing archaea in marine sponges. *Environ. Microbiol.* **10**, 1087–1094 (2008).

12. F. Hoffmann, R. Radax, D. Woebken, M. Holtappels, G. Lavik, H. T. Rapp, M.-L. Schläppy, C. Schleper, M. M. M. Kuypers, Complex nitrogen cycling in the sponge *Geodia barretti*. *Environ. Microbiol.* **11**, 2228–2243 (2009).

13. F. U. Moeller, N. S. Webster, C. W. Herbold, F. Behnam, D. Domman, M. Albertsen, M. Mooshammer, S. Markert, D. Turaev, D. Becher, T. Rattei, T. Schweder, A. Richter, M. Watzka, P. H. Nielsen, M. Wagner, Characterization of a thaumarchaeal symbiont that drives incomplete nitrification in the tropical sponge *Ianthella basta*. *Environ. Microbiol.* **21**, 3831–3854 (2019).

14. S. Zhang, W. Song, B. Wemheuer, J. Reveillaud, N. Webster, T. Thomas, Comparative genomics reveals ecological and evolutionary insights into sponge-associated Thaumarchaeota. *mSystems* **4**, e00288-19 (2019).

15. L. Moitinho-Silva, C. Díez-Vives, G. Batani, A. I. S. Esteves, M. T. Jahn, T. Thomas, Integrated metabolism in sponge-microbe symbiosis revealed by genome-centered metatranscriptomics. *ISME J.* **11**, 1651–1666 (2017).

16. B. Glasl, H. M. Luter, K. Damjanovic, K. Kitzinger, A. J. Mueller, L. Mahler, J. P. Engelberts, L. Rix, J. T. Osvatic, B. Hausmann, J. Séneca, H. Daims, P. Pjevac, M. Wagner, Co-occurring nitrifying symbiont lineages are vertically inherited and widespread in marine sponges. *ISME J.* **18**, (2024).

17. J. P. Engelberts, M. A. Abdul Wahab, M. Maldonado, L. Rix, E. Marangon, S. J. Robbins, M. Wagner, N. S. Webster, Microbes from Mum: symbiont transmission in the tropical reef sponge *Ianthella basta*. *ISME Commun* **2**, 90 (2022).

18. C. A. Francis, K. J. Roberts, J. M. Beman, A. E. Santoro, B. B. Oakley, Ubiquity and diversity of ammonia-oxidizing archaea in water columns and sediments of the ocean. *Proc. Natl. Acad. Sci. U.S.A.* **102**, 14683–14688 (2005).

19. A. N. Garritano, Z. Zhang, Y. Jia, M. A. Allen, L. J. Hill, U. Kuzhiumparambil, C. Hinkley, J. B. Raina, R. S. Peixoto, T. Thomas, Simple Porifera holobiont reveals complex interactions between the host, an archaeon, a bacterium, and a phage. *ISME J.* **18**, wrae197 (2024).

20. C. M. Preston, K. Y. Wu, T. F. Molinski, E. F. DeLong, A psychrophilic crenarchaeon inhabits a marine sponge: *Cenarchaeum symbiosum* gen. nov., sp. nov. *Proc. Natl. Acad. Sci. U.S.A.* **93**, 6241–6246 (1996).

21. K. Kitzinger, C. C. Padilla, H. K. Marchant, P. F. Hach, C. W. Herbold, A. T. Kidane, M. Könneke, S. Littmann, M. Mooshammer, J. Niggemann, S. Petrov, A. Richter, F. J. Stewart, M. Wagner, M. M. M. Kuypers, L. A. Bristow, Cyanate and urea are substrates for nitrification by Thaumarchaeota in the marine environment. *Nat. Microbiol.* **4**, 234–243 (2019).

22. M. Palatinszky, C. Herbold, N. Jehmlich, M. Pogoda, P. Han, M. von Bergen, I. Lagkouvardos, S. M. Karst, A. Galushko, H. Koch, D. Berry, H. Daims, M. Wagner, Cyanate as an energy source for nitrifiers. *Nature* **524**, 105–108 (2015).

23. J. P. Engelberts, S. J. Robbins, C. W. Herbold, F. U. Moeller, N. Jehmlich, P. W. Laffy, M. Wagner, N. S. Webster, Metabolic reconstruction of the near complete microbiome of the model sponge *Ianthella basta*. *Environ. Microbiol.* **25**, 646–660 (2023).

24. J. C. Kaiser, D. E. Heinrichs, Branching out: Alterations in bacterial physiology and virulence due to branched-chain amino acid deprivation. *mBio* **9**, e01188-18 (2018).

25. M. Maldonado, R. Aguilar, R. J. Bannister, J. J. Bell, K. W. Conway, P. K. Dayton, C. Díaz, J. Gutt, M. Kelly, E. L. R. Kenchington, S. P. Leys, S. A. Pomponi, H. T. Rapp, K. Rützler, O. S. Tendal, J. Vacelet, C. M. Young, in *Marine Animal Forests: The Ecology of Benthic Biodiversity Hotspots*, S. Rossi, L. Bramanti, A. Gori, C. Orejas, Eds. (Springer International Publishing, Cham, 2017), pp. 145–183.

26. F. O. Aylward, A. E. Santoro, Heterotrophic thaumarchaea with small genomes are widespread in the dark ocean. *mSystems* **5**, e00415-20 (2020).

27. L. Kerscher, D. Oesterhelt, The catalytic mechanism of 2-oxoacid:ferredoxin oxidoreductases from *Halobacterium halobium*. One-electron transfer at two distinct steps of the catalytic cycle. *Eur. J. Biochem.* **116**, 595–600 (1981).

28. L. Kerscher, D. Oesterhelt, Pyruvate:ferredoxin oxidoreductase—New findings on an ancient enzyme. *Trends Biochem. Sci.* **7**, 371–374 (1982).

29. G. J. Schut, A. L. Menon, M. W. W. Adams, in *Methods Enzymol* (Academic Press, 2001), vol. 331, pp. 144–158.

30. P. Reifenberg, A. Zimmer, Branched-chain amino acids: Physico-chemical properties, industrial synthesis and role in signaling, metabolism and energy production. *Amino Acids* **56**, 51 (2024).

31. S. Kim, I. Yoon, J. Son, J. Park, K. Kim, J. H. Lee, S. Y. Park, B. S. Kang, J. M. Han, K. Y. Hwang, S. Kim, Leucine-sensing mechanism of leucyl-tRNA synthetase 1 for mTORC1 activation. *Cell Rep.* **35**, 109031 (2021).

32. P. A. Voss, S. G. Gornik, M. R. Jacobovitz, S. Rupp, M. Dorr, I. Maegele, A. Guse, Host nutrient sensing is mediated by mTOR signaling in cnidarian-dinoflagellate symbiosis. *Curr. Biol.* **33**, 3634–3647.e5 (2023).

33. A. Tame, T. Maruyama, T. Ikuta, Y. Chikaraishi, N. O. Ogawa, M. Tsuchiya, K. Takishita, M. Tsuda, M. Hirai, Y. Takaki, N. Ohkouchi, K. Fujikura, T. Yoshida, mTORC1 regulates phagosome digestion of symbiotic bacteria for intracellular nutritional symbiosis in a deep-sea mussel. *Sci. Adv.* **9**, eadg8364 (2023).

34. E. B. James, H. Feng, A. C. C. Wilson, mTOR complex 1 implicated in aphid/*Buchnera* host/symbiont integration. *G3* **8**, 3083–3091 (2018).

35. S. Sogabe, N. Nakanishi, B. M. Degnan, The ontogeny of choanocyte chambers during metamorphosis in the demosponge *Amphimedon queenslandica*. *Evodevo* **7**, 6 (2016).

36. *Non-Bilaterian Phyla*, vol. 1 of *Frontiers in Invertebrate Physiology: A Collection of Reviews*, S. Saleuddin, S. P. Leys, R. D. Roer, I. C. Wilkie, Eds. (Apple Academic Press, ed. 1, 2024).

37. R. E. Sipler, D. A. Bronk, in *Biogeochemistry of Marine Dissolved Organic Matter*, D. A. Hansell, C. A. Carlson, Eds. (Academic Press, ed. 2, 2015), pp. 127–232.

38. B. Bayer, J. Vojvoda, P. Offre, R. J. E. Alves, N. H. Elisabeth, J. A. L. Garcia, J. M. Volland, M. Srivastava, J. S. P. Schleper, C. Schleper, G. J. Herndl, Physiological and genomic characterization of two novel marine thaumarchaeal strains indicates niche differentiation. *ISME J.* **10**, 1051–1063 (2016).

39. K. Kitzinger, H. K. Marchant, L. A. Bristow, C. W. Herbold, C. C. Padilla, A. T. Kidane, S. Littmann, H. Daims, P. Pjevac, F. J. Stewart, M. Wagner, M. M. M. Kuypers, Single cell analyses reveal contrasting life strategies of the two main nitrifiers in the ocean. *Nat. Commun.* **11**, 767 (2020).

40. A. E. Ingalls, S. R. Shah, R. L. Hansman, L. I. Aluwihare, G. M. Santos, E. R. Druffel, A. Pearson, Quantifying archaeal community autotrophy in the mesopelagic ocean using natural radiocarbon. *Proc. Natl. Acad. Sci. U.S.A.* **103**, 6442–6447 (2006).

41. L. M. Seyler, L. R. McGuinness, J. A. Gilbert, J. F. Biddle, D. Gong, L. J. Kerkhof, Discerning autotrophy, mixotrophy and heterotrophy in marine TACK archaea from the North Atlantic. *FEMS Microbiol. Ecol.* **94**, 10.1093/femsec/fiy014 (2018).

42. A. Pearson, A. P. McNichol, B. C. Benitez-Nelson, J. M. Hayes, T. I. Eglinton, Origins of lipid biomarkers in Santa Monica Basin surface sediment: A case study using compound-specific $\Delta^{14}C$ analysis. *Geochim. Cosmochim. Acta* **65**, 3123–3137 (2001).

43. C. Wuchter, S. Schouten, H. T. S. Boschker, J. S. Sinninghe Damsté, Bicarbonate uptake by marine Crenarchaeota. *FEMS Microbiol. Lett.* **219**, 203–207 (2003).

44. A. Vuillemin, S. D. Wankel, O. K. Coskun, T. Magritsch, S. Vargas, E. R. Estes, A. J. Spivack, D. C. Smith, R. Pockalny, R. W. Murray, S. D'Hondt, W. D. Orsi, Archaea dominate

oxic subseafloor communities over multimillion-year time scales. *Sci. Adv.* **5**, eaaw4108 (2019).

45. W. Qin, S. A. Amin, W. Martens-Habbena, C. B. Walker, H. Urakawa, A. H. Devol, A. E. Ingalls, J. W. Moffett, E. V. Armbrust, D. A. Stahl, Marine ammonia-oxidizing archaeal isolates display obligate mixotrophy and wide ecotypic variation. *Proc. Natl. Acad. Sci. U.S.A.* **111**, 12504–12509 (2014).

46. M. Tourna, M. Stieglmeier, A. Spang, M. Konneke, A. Schintlmeister, T. Urich, M. Engel, M. Schloter, M. Wagner, A. Richter, C. Schleper, *Nitrososphaera viennensis*, an ammonia oxidizing archaeon from soil. *Proc. Natl. Acad. Sci. U.S.A.* **108**, 8420–8425 (2011).

47. J.-G. Kim, S.-J. Park, J. S. S. Damsté, S. Schouten, W. I. C. Rijpstra, M.-Y. Jung, S.-J. Kim, J.-H. Gwak, H. Hong, O.-J. Si, S. Lee, E. L. Madsen, S.-K. Rhee, Hydrogen peroxide detoxification is a key mechanism for growth of ammonia-oxidizing archaea. *Proc. Natl. Acad. Sci. U.S.A.* **113**, 7888–7893 (2016).

48. B. Bayer, J. Vojvoda, T. Reinthaler, C. Reyes, M. Pinto, G. J. Herndl, *Nitrosopumilus adriaticus* sp. nov. and *Nitrosopumilus piranensis* sp. nov., two ammonia-oxidizing archaea from the Adriatic Sea and members of the class Nitrososphaeria. *Int. J. Syst. Evol. Microbiol.* **69**, 1892–1902 (2019).

49. C. C. Ouverney, J. A. Fuhrman, Marine planktonic archaea take up amino acids. *Appl. Environ. Microbiol.* **66**, 4829–4833 (2000).

50. G. J. Herndl, T. Reinthaler, E. Teira, H. van Aken, C. Veth, A. Pernthaler, J. Pernthaler, Contribution of Archaea to total prokaryotic production in the deep Atlantic Ocean. *Appl. Environ. Microbiol.* **71**, 2303–2309 (2005).

51. A. E. Dekas, A. E. Parada, X. Mayali, J. A. Fuhrman, J. Wollard, P. K. Weber, J. Pett-Ridge, Characterizing chemoautotrophy and heterotrophy in marine archaea and bacteria with single-cell multi-isotope nanoSIP. *Front. Microbiol.* **10**, 2682 (2019).

52. A. E. Parada, X. Mayali, P. K. Weber, J. Wollard, A. E. Santoro, J. A. Fuhrman, J. Pett-Ridge, A. E. Dekas, Constraining the composition and quantity of organic matter used by abundant marine Thaumarchaeota. *Environ. Microbiol.* **25**, 689–704 (2023).

53. R. Caspi, R. Billington, I. M. Keseler, A. Kothari, M. Krummenacker, P. E. Midford, W. K. Ong, S. Paley, P. Subhraveti, P. D. Karp, The MetaCyc database of metabolic pathways and enzymes—A 2019 update. *Nucleic Acids Res.* **48**, D445–D453 (2020).

54. E. L. Clifford, D. De Corte, C. Amano, P. Paliaga, I. Ivancic, V. Ortiz, M. Najdek, G. J. Herndl, E. Sintes, Mesozooplankton taurine production and prokaryotic uptake in the northern Adriatic Sea. *Limnol. Oceanogr.* **65**, 2730–2747 (2020).

55. P. F. Langenbruch, W. C. Jones, Body structure of marine sponges. VI. Choanocyte chamber structure in the haplosclerida (porifera, demospongiae) and its relevance to the phylogenesis of the group. *J. Morphol.* **204**, 1–8 (1990).

56. J. M. De Goeij, A. De Kluijver, F. C. Van Duyl, J. Vacelet, R. H. Wijffels, A. F. De Goeij, J. P. Cleutjens, B. Schutte, Cell kinetics of the marine sponge *Halisarca caerulea* reveal rapid cell turnover and shedding. *J. Exp. Biol.* **212**, 3892–3900 (2009).

57. M. Hudspith, L. Rix, M. Achlatis, J. Bougoure, P. Guagliardo, P. L. Clode, N. S. Webster, G. Muyzer, M. Pernice, J. M. de Goeij, Subcellular view of host-microbiome nutrient exchange in sponges: Insights into the ecological success of an early metazoan-microbe symbiosis. *Microbiome* **9**, 44 (2021).

58. D. H. Parks, M. Chuvochina, D. W. Waite, C. Rinke, A. Skarshewski, P. A. Chaumeil, P. Hugenholtz, A standardized bacterial taxonomy based on genome phylogeny substantially revises the tree of life. *Nat. Biotechnol.* **36**, 996–1004 (2018).

59. D. H. Parks, M. Imelfort, C. T. Skennerton, P. Hugenholtz, G. W. Tyson, CheckM: Assessing the quality of microbial genomes recovered from isolates, single cells, and metagenomes. *Genome Res.* **25**, 1043–1055 (2015).

60. B. Q. Minh, H. A. Schmidt, O. Chernomor, D. Schrempf, M. D. Woodhams, A. von Haeseler, R. Lanfear, IQ-TREE 2: New models and efficient methods for phylogenetic inference in the genomic era. *Mol. Biol. Evol.* **37**, 1530–1534 (2020).

61. I. Letunic, P. Bork, Interactive Tree of Life (iTOL) v6: Recent updates to the phylogenetic tree display and annotation tool. *Nucleic Acids Res.* **52**, W78–W82 (2024).

62. C. P. Cantalapiedra, A. Hernandez-Plaza, I. Letunic, P. Bork, J. Huerta-Cepas, eggNOG-mapper v2: Functional annotation, orthology assignments, and domain prediction at the metagenomic scale. *Mol. Biol. Evol.* **38**, 5825–5829 (2021).

63. M. D. Adams, L. M. Wagner, T. J. Graddis, R. Landick, T. K. Antonucci, A. L. Gibson, D. L. Oxender, Nucleotide sequence and genetic characterization reveal six essential genes for the LIV-I and LS transport systems of *Escherichia coli*. *J. Biol. Chem.* **265**, 11436–11443 (1990).

64. D. R. Speth, N. Pullen, S. T. N. Aroney, B. L. Coltman, J. Osvatic, B. J. Woodcroft, T. Rattei, M. Wagner, GlobDB: A comprehensive species-dereplicated microbial genome resource. *Bioinform. Adv.* **5**, vbaf280 (2025).

65. W. Shen, B. Sipos, L. Zhao, SeqKit2: A Swiss army knife for sequence and alignment processing. *Imeta* **3**, e191 (2024).

66. R. C. Edgar, Search and clustering orders of magnitude faster than BLAST. *Bioinformatics* **26**, 2460–2461 (2010).

67. R. C. Edgar, Muscle5: High-accuracy alignment ensembles enable unbiased assessments of sequence homology and phylogeny. *Nat. Commun.* **13**, 6968 (2022).

68. M. N. Price, P. S. Dehal, A. P. Arkin, FastTree 2—Approximately maximum-likelihood trees for large alignments. *PLOS ONE* **5**, e9490 (2010).

69. T. Brůna, K. J. Hoff, A. Lomsadze, M. Stanke, M. Borodovsky, BRAKER2: Automatic eukaryotic genome annotation with GeneMark-EP+ and AUGUSTUS supported by a protein database. *NAR Genom. Bioinform.* **3**, lqaa108 (2021).

70. V. McKenna, J. M. Archibald, R. Beinart, M. N. Dawson, U. Hentschel, P. J. Keeling, J. V. Lopez, J. M. Martin-Duran, J. M. Petersen, J. D. Sigwart, O. Simakov, K. R. Sutherland, M. Sweet, N. J. Talbot, A. W. Thompson, S. Bender, P. W. Harrison, J. Rajan, G. Cochrane, M. Berriman, M. K. N. Lawniczak, M. Blaxter, The Aquatic Symbiosis Genomics Project: Probing the evolution of symbiosis across the Tree of Life. *Wellcome Open Res* **6**, 254 (2021).

71. M. Kanehisa, Y. Sato, K. Morishima, BlastKOALA and GhostKOALA: KEGG tools for functional characterization of genome and metagenome sequences. *J. Mol. Biol.* **428**, 726–731 (2016).

72. E. Salas, M. Gorfer, D. Bandian, B. Wang, C. Kaiser, W. Wanek, A rapid and sensitive assay to quantify amino sugars, neutral sugars and uronic acid necromass biomarkers using pre-column derivatization, ultra-high-performance liquid chromatography and high-resolution mass spectrometry. *Soil Biol. Biochem.* **177**, 108927 (2023).

73. K. M. Miranda, M. G. Espey, D. A. Wink, A rapid, simple spectrophotometric method for simultaneous detection of nitrate and nitrite. *Nitric. Oxide* **5**, 62–71 (2001).

74. E. García-Robledo, A. Corzo, S. Papaspyrou, A fast and direct spectrophotometric method for the sequential determination of nitrate and nitrite at low concentrations in small volumes. *Mar. Chem.* **162**, 30–36 (2014).

75. M. R. McIlvin, M. A. Altabet, Chemical conversion of nitrate and nitrite to nitrous oxide for nitrogen and oxygen isotopic analysis in freshwater and seawater. *Anal. Chem.* **77**, 5589–5595 (2005).

76. A. E. Parada, D. M. Needham, J. A. Fuhrman, Every base matters: Assessing small subunit rRNA primers for marine microbiomes with mock communities, time series and global field samples. *Environ. Microbiol.* **18**, 1403–1414 (2016).

77. A. Apprill, S. McNally, R. Parsons, L. Weber, Minor revision to V4 region SSU rRNA 806R gene primer greatly increases detection of SAR11 bacterioplankton. *Aquat. Microb. Ecol.* **75**, 129–137 (2015).

78. P. Pjevac, B. Hausmann, J. Schwarz, G. Kohl, C. W. Herbold, A. Loy, D. Berry, An economical and flexible dual barcoding, two-step PCR approach for highly multiplexed amplicon sequencing. *Front. Microbiol.* **12**, 669776 (2021).

79. B. J. Callahan, P. J. McMurdie, M. J. Rosen, A. W. Han, A. J. A. Johnson, S. P. Holmes, DADA2: High-resolution sample inference from Illumina amplicon data. *Nat. Methods* **13**, 581–583 (2016).

80. R Development Core Team, R: A language and environment for statistical computing. (R Foundation for Statistical Computing, 2008).

81. P. J. McMurdie, S. Holmes, phyloseq: An R package for reproducible interactive analysis and graphics of microbiome census data. *PLOS ONE* **8**, e61217 (2013).

82. H. Wickham, R. François, L. Henry, K. Müller, D. Vaughan, dplyr (2023); https://dplyr.tidyverse.org.

83. H. Wickham, *ggplot2: Elegant Graphics for Data Analysis* (Springer, 2009).

84. A. Pernthaler, J. Pernthaler, R. Amann, in *Molecular Microbial Ecology Manual*, G. Kowalchuk, Ed. (Kluwer Academic Publishers, 2004), chap. 3.11, pp. 711–726.

85. D. A. Stahl, R. Amann, in *Nucleic Acid Techniques in Bacterial Systematics*, E. Stackebrandt, M. Goodfellow, Eds. (John Wiley and Sons Ltd., 1991), pp. 205–248.

86. F. U. Moeller, C. W. Herbold, A. Schintlmeister, M. Mooshammer, C. Motti, B. Glasl, K. Kitzinger, F. Behnam, M. Watzka, T. Schweder, M. Albertsen, A. Richter, N. S. Webster, M. Wagner, Taurine as a key intermediate for host-symbiont interaction in the tropical sponge *Ianthella basta*. *ISME J.* **17**, 1208–1223 (2023).

87. G. Wallner, R. Amann, W. Beisker, Optimizing fluorescent in situ hybridization with rRNA-targeted oligonucleotide probes for flow cytometric identification of microorganisms. *Cytometry* **14**, 136–143 (1993).

88. A. A. Legin, A. Schintlmeister, N. S. Sommerfeld, M. Eckhard, S. Theiner, S. Reipert, D. Strohhofer, M. A. Jakupec, M. S. Galanski, M. Wagner, B. K. Keppler, Nano-scale imaging of dual stable isotope labeled oxaliplatin in human colon cancer cells reveals the nucleolus as a putative node for therapeutic effect. *Nanoscale Adv.* **3**, 249–262 (2021).

89. N. Musat, H. Stryhanyuk, P. Bombach, L. Adrian, J.-N. Audinot, H. H. Richnow, The effect of FISH and CARD-FISH on the isotopic composition of ^{13}C- and ^{15}N-labeled *Pseudomonas putida* cells measured by NanoSIMS. *Syst. Appl. Microbiol.* **37**, 267–276 (2014).

90. D. Woebken, L. C. Burow, F. Behnam, X. Mayali, A. Schintlmeister, E. D. Fleming, L. Prufert-Bebout, S. W. Singer, A. L. Cortes, T. M. Hoehler, J. Pett-Ridge, A. M. Spormann, M. Wagner, P. K. Weber, B. M. Bebout, Revisiting N_2 fixation in Guerrero Negro intertidal microbial mats with a functional single-cell approach. *ISME J.* **9**, 485–496 (2015).

91. N. R. Meyer, J. L. Fortney, A. E. Dekas, NanoSIMS sample preparation decreases isotope enrichment: magnitude, variability and implications for single-cell rates of microbial activity. *Environ. Microbiol.* **23**, 81–98 (2021).

92. C. Martínez-Pérez, W. Mohr, C. R. Löscher, J. Dekaezemacker, S. Littmann, P. Yilmaz, N. Lehnen, B. M. Fuchs, G. Lavik, R. A. Schmitz, J. LaRoche, M. M. M. Kuypers, The small unicellular diazotrophic symbiont, UCYN-A, is a key player in the marine nitrogen cycle. *Nat. Microbiol.* **1**, 16163 (2016).

93. A. Krupke, W. Mohr, J. LaRoche, B. M. Fuchs, R. I. Amann, M. M. M. Kuypers, The effect of nutrients on carbon and nitrogen fixation by the UCYN-A-haptophyte symbiosis. *ISME J.* **9**, 1635–1647 (2015).

94. A. Khachikyan, J. Milucka, S. Littmann, S. Ahmerkamp, T. Meador, M. Konneke, T. Burg, M. M. M. Kuypers, Direct cell mass measurements expand the role of small microorganisms in nature. *Appl. Environ. Microbiol.* **85**, e00493-19 (2019).

95. J. Sun, D. Liu, Geometric models for calculating cell biovolume and surface area for phytoplankton. *J. Plankton Res.* **25**, 1331–1346 (2003).

96. M. Konneke, D. M. Schubert, P. C. Brown, M. Hugler, S. Standfest, T. Schwander, L. Schada von Borzyskowski, T. J. Erb, D. A. Stahl, I. A. Berg, Ammonia-oxidizing archaea use the most energy-efficient aerobic pathway for CO_2 fixation. *Proc. Natl. Acad. Sci. U.S.A.* **111**, 8239–8244 (2014).

97. J. Pinhassi, M. J. Pujalte, J. Pascual, J. M. González, I. Lekunberri, C. Pedrós-Alió, D. R. Arahal, *Bermanella marisrubri* gen. nov., sp. nov., a genome-sequenced gammaproteobacterium from the Red Sea. *Int. J. Syst. Evol. Microbiol.* **59**, 373–377 (2009).
